# Ceftolozane/tazobactam versus meropenem in patients with ventilated hospital-acquired bacterial pneumonia: subset analysis of the ASPECT-NP randomized, controlled phase 3 trial

**DOI:** 10.1186/s13054-021-03694-3

**Published:** 2021-08-11

**Authors:** Jean-François Timsit, Jennifer A. Huntington, Richard G. Wunderink, Nobuaki Shime, Marin H. Kollef, Ülo Kivistik, Martin Nováček, Álvaro Réa-Neto, Ignacio Martin-Loeches, Brian Yu, Erin H. Jensen, Joan R. Butterton, Dominik J. Wolf, Elizabeth G. Rhee, Christopher J. Bruno

**Affiliations:** 1 Intensive Care Medicine Department, Université Paris Diderot, Paris, France; 2 MRL, Merck & Co., Inc., Kenilworth, NJ USA; 3 Pulmonary and Critical Care Division, Northwestern University Feinberg School of Medicine, Chicago, IL USA; 4 Department of Emergency and Critical Care Medicine, Hiroshima University, Hiroshima, Japan; 5 Division of Pulmonary and Critical Care Medicine, Washington University School of Medicine, St. Louis, MO USA; 6 Pulmonology Centre, North Estonia Medical Centre, Tallinn, Estonia; 7 Department of Anaesthesia and Intensive Care, General Hospital of Kolin, Kolin, Czech Republic; 8 Departamento de Clínica Médica, Universidade Federal do Paraná, Curitiba, Brazil; 9 Department of Intensive Care Medicine, Multidisciplinary Intensive Care Research Organization (MICRO), St James’ Hospital, Dublin, Ireland; 10 Hospital Clinic, Universitat de Barcelona, IDIBAPS, CIBERES, Barcelona, Spain

**Keywords:** Nosocomial pneumonia, HABP/VABP, Mechanical ventilation, *Pseudomonas aeruginosa*, ESBL, All-cause mortality, Clinical response, Multivariable analysis

## Abstract

**Background:**

Ceftolozane/tazobactam is approved for treatment of hospital-acquired/ventilator-associated bacterial pneumonia (HABP/VABP) at double the dose approved for other infection sites. Among nosocomial pneumonia subtypes, ventilated HABP (vHABP) is associated with the lowest survival. In the ASPECT-NP randomized, controlled trial, participants with vHABP treated with ceftolozane/tazobactam had lower 28-day all-cause mortality (ACM) than those receiving meropenem. We conducted a series of post hoc analyses to explore the clinical significance of this finding.

**Methods:**

ASPECT-NP was a multinational, phase 3, noninferiority trial comparing ceftolozane/tazobactam with meropenem for treating vHABP and VABP; study design, efficacy, and safety results have been reported previously. The primary endpoint was 28-day ACM. The key secondary endpoint was clinical response at test-of-cure. Participants with vHABP were a prospectively defined subgroup, but subgroup analyses were not powered for noninferiority testing. We compared baseline and treatment factors, efficacy, and safety between ceftolozane/tazobactam and meropenem in participants with vHABP. We also conducted a retrospective multivariable logistic regression analysis in this subgroup to determine the impact of treatment arm on mortality when adjusted for significant prognostic factors.

**Results:**

Overall, 99 participants in the ceftolozane/tazobactam and 108 in the meropenem arm had vHABP. 28-day ACM was 24.2% and 37.0%, respectively, in the intention-to-treat population (95% confidence interval [CI] for difference: 0.2, 24.8) and 18.2% and 36.6%, respectively, in the microbiologic intention-to-treat population (95% CI 2.5, 32.5). Clinical cure rates in the intention-to-treat population were 50.5% and 44.4%, respectively (95% CI − 7.4, 19.3). Baseline clinical, baseline microbiologic, and treatment factors were comparable between treatment arms. Multivariable regression identified concomitant vasopressor use and baseline bacteremia as significantly impacting ACM in ASPECT-NP; adjusting for these two factors, the odds of dying by day 28 were 2.3-fold greater when participants received meropenem instead of ceftolozane/tazobactam.

**Conclusions:**

There were no underlying differences between treatment arms expected to have biased the observed survival advantage with ceftolozane/tazobactam in the vHABP subgroup. After adjusting for clinically relevant factors found to impact ACM significantly in this trial, the mortality risk in participants with vHABP was over twice as high when treated with meropenem compared with ceftolozane/tazobactam.

***Trial registration*:**

clinicaltrials.gov, NCT02070757. Registered 25 February, 2014, clinicaltrials.gov/ct2/show/NCT02070757.

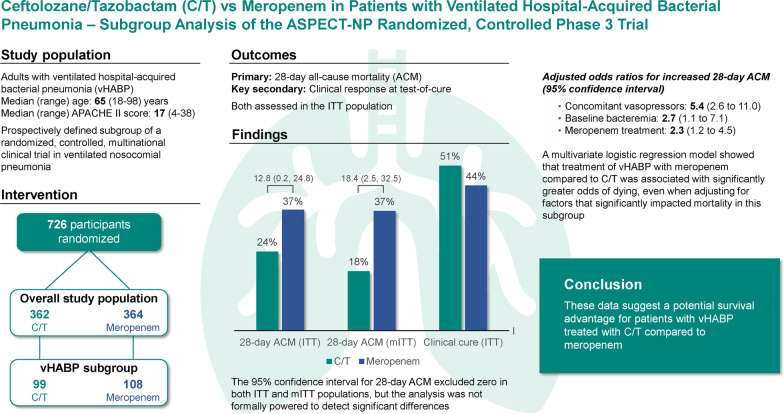

**Supplementary Information:**

The online version contains supplementary material available at 10.1186/s13054-021-03694-3.

## Background

Pneumonia, including hospital-acquired and ventilator-associated bacterial pneumonia (HABP/VABP), is the most common healthcare-acquired infection in the intensive care unit and is associated with high mortality rates of ~ 20–50% [1–3]. HABP is classified as bacterial pneumonia that develops in patients who have been hospitalized for ≥ 48 h, while VABP is defined as bacterial pneumonia developing after ≥ 48 h of endotracheal intubation [2–4]. Patients with HABP who progress to respiratory failure severe enough to require mechanical ventilation (vHABP) represent a more clinically severe subtype of the disease than patients with nonventilated HABP [4]. Randomized controlled trials evaluating antibacterial treatment of HABP/VABP consistently demonstrate higher mortality in participants with vHABP compared with VABP or nonventilated HABP [1, 5]. Clinical research in nosocomial pneumonia has tended to focus more on VABP than HABP, with even less focus specifically on the vHABP subgroup. The limited number of available published studies do not report substantial differences between VABP and HABP in terms of underlying microbiology or pathophysiology [4, 6]. Given the especially high mortality associated with vHABP, new treatment options are particularly needed for this nosocomial pneumonia subtype [1].

Ceftolozane/tazobactam is a combination antibacterial agent comprising ceftolozane, an anti-pseudomonal cephalosporin, and tazobactam, a β-lactamase inhibitor active against extended-spectrum β-lactamases (ESBLs) [7]. The combination has broad in vitro activity against gram-negative lower respiratory tract (LRT) pathogens, including multidrug-resistant *P. aeruginosa* and ESBL-producing Enterobacterales, but lacks activity against strains expressing metallo-β-lactamases (MBLs) and other carbapenemases [8–11]. Ceftolozane/tazobactam has also demonstrated good intrapulmonary penetration in two clinical trials, including a study conducted exclusively in critically ill, ventilated participants [12]. Ceftolozane/tazobactam was recently approved for the treatment of HABP/VABP in adults using a regimen of 3 g (2 g ceftolozane and 1 g tazobactam) every 8 h (q8h), which is double the dose indicated for complicated intrabdominal and complicated urinary tract infections [13]. This approval was based on results from the ASPECT-NP phase 3 trial, which demonstrated ceftolozane/tazobactam to be noninferior to meropenem for treating HABP/VABP in both primary and key secondary endpoints [14]. In participants with vHABP, the higher mortality expected compared with VABP was only seen with meropenem (37% vHABP, 20% VABP) but not ceftolozane/tazobactam (24% in both vHABP and VABP) [14].

To explore the potential survival advantage with ceftolozane/tazobactam in this particular patient population further, we evaluated efficacy and safety outcomes specifically in ASPECT-NP participants with vHABP.

## Methods

### Overall clinical trial design

ASPECT-NP was a phase 3, randomized, controlled, double-blind, multicenter, noninferiority trial comparing ceftolozane/tazobactam with meropenem for the treatment of vHABP/VABP. Briefly, ASPECT-NP enrolled intubated, mechanically ventilated patients ≥ 18 years old with VABP or vHABP, diagnosed based on stringent clinical and radiographic criteria, within 24 h prior to first dose of study drug [15]. The diagnostic criteria for vHABP required at least one of the following signs and/or symptoms (present within 24 h prior to intubation or within 48 h after intubation) in patients either hospitalized for ≥ 48 h or discharged from a hospital within ≤ 7 days (including patients in skilled nursing or other long-term care facilities): new onset of cough or worsening of baseline cough; dyspnea, tachypnea, or respiratory rate > 30/min, particularly if progressive in nature; and/or hypoxemia (arterial blood gas partial pressure of oxygen < 60 mmHg while breathing room air, or a pulse oximetry oxygen saturation < 90% while breathing room air, or worsening of PaO_2_/FiO_2_). In addition, eligible patients needed the following within ≤ 24 h of first dose of study drug: a chest radiograph or CT scan showing new or progressive infiltrate(s) suggestive of bacterial pneumonia and at least one of the following: fever (body temperature ≥ 38 °C), hypothermia (body temperature ≤ 35 °C), white blood cell count ≤ 4500 cells/mm^3^ or ≥ 10,000 cells/mm^3^, or ≥ 15% immature neutrophils. Potential participants with any of the following were ineligible: baseline Gram stain with only gram-positive pathogens; > 24 h of active, systemic, or inhaled gram-negative antibacterial agents for HABP/VABP within 72 h prior (unless patients were failing such therapy at the time of enrolment); meropenem- or ceftolozane/tazobactam-nonsusceptible, gram-negative pathogen(s) isolated from respiratory or blood cultures ≤ 15 days prior; known/suspected community-acquired pneumonia; nonbacterial pneumonia; active immunosuppression and/or neutropenia; continuous renal replacement therapy; or end-stage renal disease requiring hemodialysis.

Eligible participants were randomized 1:1 to either 3 g q8h ceftolozane/tazobactam or 1 g q8h meropenem, stratified by diagnosis (VABP vs vHABP) and age (< 65 vs ≥ 65 years). Investigators, study staff (except pharmacists preparing masked infusion bags), and participants/participant representatives remained blinded to treatment throughout the study. In participants with moderate or severe renal impairment, the unblinded pharmacist adjusted dosing of randomized study drug according to the approved regimen. Total duration of study treatment was 8–14 d, with 14 days recommended for *P. aeruginosa* infections. Adjunctive linezolid 600 mg IV q12h was required for all participants until baseline LRT cultures confirmed absence of *Staphylococcus aureus*; adjunctive empiric therapy with amikacin 15 mg/kg was permitted for the first 72 h of study treatment at hospitals where ≥ 15% of *Pseudomonas aeruginosa* isolates are known to be meropenem resistant. If any other non-study HABP/VABP treatment was required after randomization due to clinical or microbiologic failure, study drug was discontinued. A baseline LRT specimen was required from all participants within 36 h prior to randomization. Post-baseline LRT cultures were collected from still intubated participants during the first week of treatment and at end-of-therapy (EOT) and test-of-cure (TOC; 7–14 days post-EOT). Susceptibility to ceftolozane/tazobactam was determined using the following breakpoints: ≤ 4 μg/mL for Enterobacterales and ≤ 8 μg/mL for *P. aeruginosa*, *Acinetobacter baumannii*, *Haemophilus influenzae*, and other bacterial pathogens. Meropenem susceptibility was determined based on current Clinical and Laboratory Standards Institute (CLSI) breakpoints [16]. In this trial, microbiology data were only collected for gram-negative and streptococcal LRT pathogens. Of note, like meropenem, ceftolozane/tazobactam is not active against pathogens expressing MBLs.

The primary endpoint was 28-day all-cause mortality (ACM) and the key secondary endpoint was clinical response at TOC, both assessed in the intent-to-treat (ITT) population comprising all randomized participants. Other secondary endpoints included clinical response at TOC in the clinically evaluable (CE) population (defined as participants who received study drug, adhered to the study protocol, and had an evaluable clinical outcome at TOC), per-pathogen microbiologic response and per-patient microbiologic response at TOC in the microbiologic ITT (mITT) population (defined as participants with ≥ 1 dose of study treatment and ≥ 1 gram-negative or streptococcal respiratory pathogen from baseline LRT cultures that was susceptible to ≥ 1 study drug) and in the microbiologically evaluable (ME) population (defined as participants who received study drug, adhered to the study protocol, had ≥ 1 gram-negative or streptococcal respiratory pathogen from baseline LRT cultures [at the appropriate colony-forming unit (CFU)/mL threshold: ≥ 10^5^ CFU/mL for endotracheal aspiration, ≥ 10^4^ CFU/mL for bronchoalveolar lavage/mini-bronchoalveolar lavage, and ≥ 10^3^ CFU/mL for protected specimen brush] from the baseline LRT culture that was susceptible to ≥ 1 study drug, and had an evaluable clinical outcome at TOC), and 28-day ACM in the mITT population. Safety was assessed from first dose of study treatment to the late follow-up (LFU) visit (28–35 days after EOT) in all randomized participants who received ≥ 1 dose of study treatment, according to actual treatment received.

### Subgroup analyses

vHABP and VABP were prospectively defined patient subgroups and stratification factors for randomization. A vHABP diagnosis required participants to meet all of the following criteria (see above for details): intubation and mechanical ventilation; predefined clinical and radiographic criteria for a pneumonia diagnosis; hospitalization for ≥ 48 h or hospital discharge ≤ 7 days prior to diagnosis (including participants institutionalized in skilled nursing or other long-term care facility). We compared baseline clinical and microbiologic factors, treatment factors, efficacy, and safety between treatment arms specifically in participants with vHABP.

The primary and key secondary efficacy endpoints were prospectively evaluated in both the vHABP and VABP subgroups, while all other analyses were conducted retrospectively. These subgroup analyses were not powered for noninferiority testing, and no stratification was applied; 95% confidence intervals (CIs) associated with the treatment differences were calculated as unstratified Newcombe CIs [17]. For analyses in the ITT and mITT populations, missing responses (including indeterminates) were considered deaths or clinical or microbiologic failures (depending on the specific analysis); for CE and ME analyses, all missing responses were excluded from analysis. Safety data were analyzed descriptively. All statistical analyses were conducted using SAS version 9.3 (SAS Institute, Cary, NC).

### Multivariable analysis

A multiple logistic regression analysis (Additional file 1: Figure S1) was performed to evaluate two questions: (1) which clinical factors predicted 28-day ACM in the vHABP subgroup of this trial, and (2) whether treatment arm assignment still impacted 28-day ACM after adjusting for any significant predictive factors. Sixteen clinical and microbiologic factors with the potential to affect treatment outcomes and which were available from the collected data were prospectively selected for evaluation in this analysis: age, creatinine clearance (CrCL), APACHE-II score, Sequential Organ Failure Assessment (SOFA) score, Clinical Pulmonary Infection Score (CPIS), and PaO_2_/FiO_2_ were assessed as continuous variables; failed antibacterial therapy for the current pneumonia episode, ≥ 5 days prior hospitalization, ≥ 5 days prior mechanical ventilation, baseline bacteremia (any pathogen), all baseline pathogens susceptible to randomized study drug, baseline *P. aeruginosa*, baseline ESBL-positive Enterobacterales, adjunctive gram-negative therapy, concomitant vasopressor use, and treatment arm (ceftolozane/tazobactam vs meropenem) were assessed as categorical, dichotomous variables (presence vs absence).

Since several of the selected factors are known to have multicollinearity, the methodology used to identify significant explanatory variables for inclusion into the multivariable model needed to be chosen carefully. For this reason, we used the random forest ensemble method [18, 19], a type of decision-tree learning algorithm that is able to address nonlinear relationships and complex interactions between potential explanatory variables, to build a classification ranking the relative importance of each factor in predicting mortality. The random forest analysis was conducted using the package randomForest (CRAN; version 4.6–14) in R (CRAN; version 3.6.6). The original dataset for the random forest analysis was the full vHABP ITT analysis population from ASPECT-NP. The random forest algorithm constructs multiple decision trees using bootstrap aggregation; i.e., for each tree, input data (referred to as ‘training data’) is randomly resampled with replacement from the overall dataset. The algorithm then calculates a consensus predictive model from the combined results of all the individual decision trees. For each model run, the algorithm randomly excludes approximately one-third of the original dataset from model training, instead using it to evaluate model performance and thus avoiding the need for a separate validation dataset. These evaluation data are referred to as the out of bag (OOB) sample. The model error that is estimated on the OOB samples is used to assess the predictive capability of the factors (i.e., how strongly each factor impacted 28-day ACM). Within each model run, the relative importance of all factors was then ranked from most to least important. A total of 200 model runs were completed, and the ranking of the 16 preselected factors was then used as the order in which factors were added to the logistic regression model using forward variable selection (i.e., the most important factor was included into the model first, with the other factors being included one-by-one in the order of their ranking). The area under the receiver operating characteristic (ROC) curve was estimated at each step of forward variable selection and was used as the metric to assess which factors were most influential in model prediction.

Factors identified as important during forward variable selection were then further explored using a backwards selection logistic regression model, in order to determine their impact on 28-day ACM in the vHABP subgroup. Treatment group was included into this regression model irrespective of results of the previous step, since the goal of these analyses was to determine the impact of treatment assignment on mortality relative to the impact of the most influential factors. The backwards elimination logistic regression model was fit with main effects for all included variables; interaction effects were not included. All terms with a p-value of < 0.05 remained in the backwards selection logistic regression model. Odd ratios (ORs) and 95% CIs, representing the increase in the odds of 28-day ACM, were estimated from the final model. We also conducted a traditional multivariable logistic regression as a sensitivity analysis, in which all 16 of the selected factors were included as main effects. All logistic regression analyses were performed using SAS version 9.3 (SAS Institute, Cary, NC).

## Results

### Patients

**Fig. 1:**
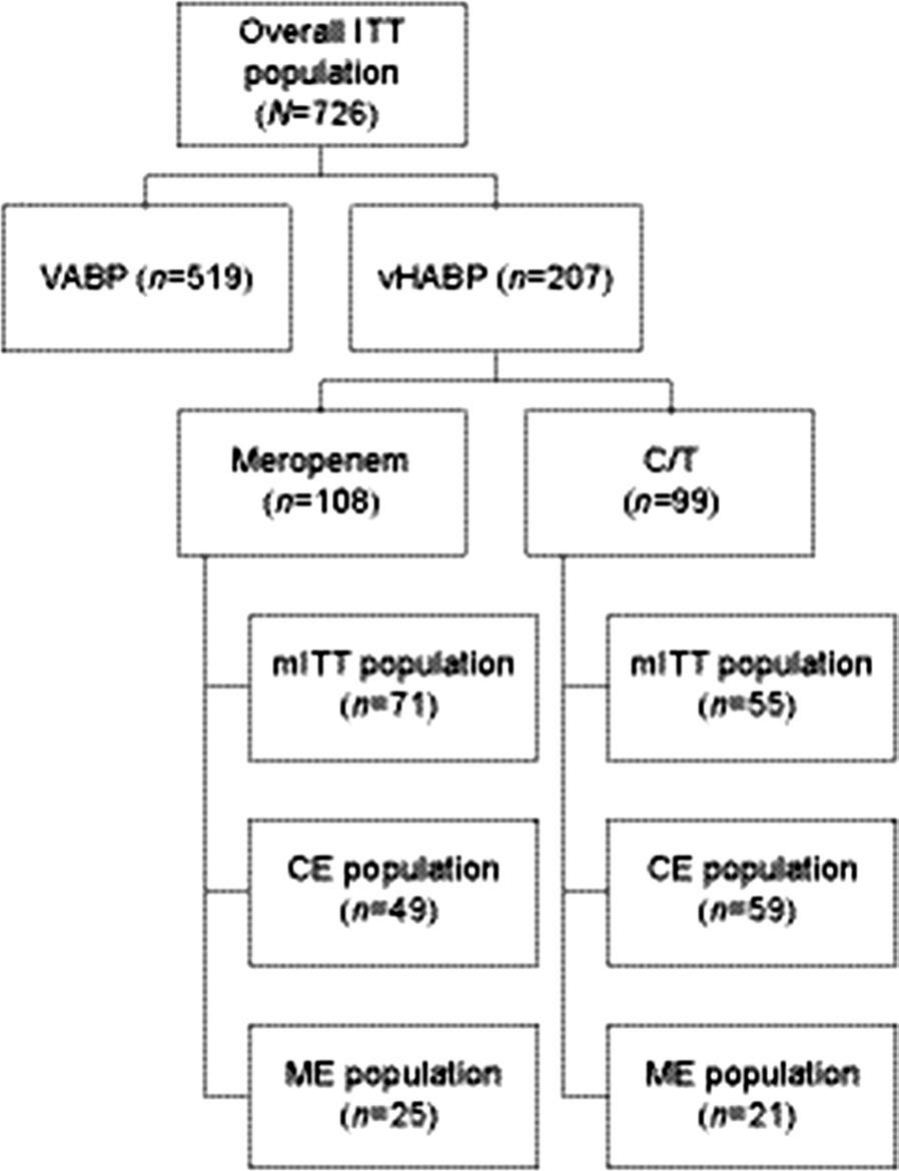
Patient and analysis population flow chart. CE, clinically evaluable. C/T, ceftolozane/tazobactam. ITT, intent-to-treat. ME, microbiologically evaluable. mITT, microbiologic intent-to-treat. N, number of participants in specific analysis population. VABP, ventilator-associated bacterial pneumonia. vHABP, ventilated hospital-acquired pneumonia

In the ITT primary efficacy population, 99/362 (27.3%) ceftolozane/tazobactam and 108/364 (29.7%) meropenem participants had vHABP (Fig. 1). Compared to participants with VABP, those with vHABP were more likely to be elderly, be renally impaired, have SOFA scores > 7, and have failed antibacterial therapy for their current pneumonia episode, and they were less likely to have augmented renal clearance (ARC) or to have been hospitalized ≥ 5 days (Additional file 1: Table S1). Within the subgroup of vHABP participants, baseline demographics and clinical characteristics were generally comparable between treatment arms (Table 1). Participants in the ceftolozane/tazobactam arm were more likely (≥ 5% difference) to have moderate renal impairment, received prior antibacterial therapy active against gram-negative pathogens in the 72 h prior to first dose of study drug, and to have been hospitalized for ≥ 5 days, while participants in the meropenem arm were more frequently elderly and had CPIS > 8. Concomitant vasopressors (i.e., adrenergic or dopaminergic agents administered between the first dose of study drug and LFU) were reported for 55/99 (55.6%) ceftolozane/tazobactam- and 55/108 (50.9%) meropenem-treated participants with vHABP.

**Table 1:** Baseline demographics and clinical characteristics in ASPECT-NP participants with ventilated hospital-acquired bacterial pneumonia (ITT population). Prognostic factors expected to lead to worse clinical outcomes are in bold text

	C/T (*N* = 99)	Meropenem (*N* = 108)	Total (*N* = 207)
*Sex*			
Male, *n* (%)	74 (74.7)	72 (66.7)	146 (70.5)
Female, *n* (%)	25 (25.3)	36 (33.3)	61 (29.5)
*Age, years*			
< 65, *n* (%)	52 (52.5)	47 (43.5)	99 (47.8)
**≥ 65, *n* (%)**	47 (47.5)	61 (56.5)	108 (52.2)
Mean (standard deviation)	63.3 (15.0)	65.3 (14.7)	64.3 (14.9)
Median (range)	64.0 (18, 98)	66.5 (24, 92)	65.0 (18, 98)
*Weight, kg*			
Median (range)	78.1 (34.0, 140.0)	75.0 (40.0, 151.0)	78.0 (34.0, 151.0)
*Body-mass index, kg*			
Median (range)	26.2 (15.1, 44.1)	26.1 (15.5, 49.3)	26.1 (15.1, 49.3)
*Creatinine clearance (mL/min)*			
**≥ 150 (augmented renal clearance), *n* (%)**	9 (9.1)	5 (4.6)	14 (6.8)
≥ 80, *n* (%)	44 (44.4)	48 (44.4)	92 (44.4)
< 80 to > 50, *n* (%)	26 (26.3)	34 (31.5)	60 (29.0)
**≤ 50 to ≥ 30, *n* (%)**	17 (17.2)	10 (9.3)	27 (13.0)
**< 30 to ≥ 15, *n* (%)**	12 (12.1)	13 (12.0)	25 (12.1)
**< 15 (end-stage renal disease), *n* (%)**	0	0	0
Missing, *n* (%)	0	3 (2.8)	3 (1.4)
*In the ICU*			
**Yes, *n* (%)**	83 (83.8)	86 (79.6)	169 (81.6)
No, *n* (%)	16 (16.2)	22 (20.4)	38 (18.4)
*APACHE II score*			
≤ 14, *n* (%)	32 (32.3)	27 (25.0)	59 (28.5)
15–19, *n* (%)	36 (36.4)	40 (37.0)	76 (36.7)
**≥ 20, *n* (%)**	31 (31.3)	40 (37.0)	71 (34.3)
Missing, *n* (%)	0	1 (0.9)	1 (0.5)
Mean (standard deviation)	17.5 (6.1)	18.4 (5.9)	18.0 (6.0)
Median (range)	16.0 (4, 33)	17.0 (5, 38)	17.0 (4, 38)
*SOFA score*			
≤ 7, *n* (%)	57 (57.6)	65 (60.2)	122 (58.9)
**> 7, *n* (%)**	42 (42.4)	41 (38.0)	83 (40.1)
Missing, *n* (%)	0	2 (1.9)	2 (1.0)
Mean (standard deviation)	7.2 (2.7)	7.1 (2.7)	7.1 (2.7)
Median (range)	7 (0, 13)	7 (1, 15)	7 (0, 15)
Prior non-study gram-negative therapy^*^			
**Yes, *n* (%)**	92 (92.9)	93 (86.1)	185 (89.4)
No, *n* (%)	7 (7.1)	15 (13.9)	22 (10.6)
*Failed prior antibacterial therapy for NP*			
**Yes, *n* (%)**	20 (20.2)	17 (15.7)	37 (17.9)
No, *n* (%)	79 (79.8)	90 (83.3)	169 (81.6)
Missing, *n* (%)	0	1 (0.9)	1 (0.5)
*Adjunctive gram-negative therapy*^§^			
Yes, *n* (%)	35 (35.4)	34 (31.5)	69 (33.3)
No, *n* (%)	64 (64.6)	70 (64.8)	134 (64.7)
Missing, *n* (%)	0	4 (3.7)	4 (1.9)
*CPIS*			
≤ 6, *n* (%)	9 (9.1)	7 (6.5)	16 (7.7)
7, *n* (%)	12 (12.1)	7 (6.5)	19 (9.2)
**8, *n* (%)**	13 (13.1)	13 (12.0)	26 (12.6)
**> 8, *n* (%)**	65 (65.7)	80 (74.1)	145 (70.0)
Missing, *n* (%)	0	1 (0.9)	1 (0.5)
*Duration of prior hospitalization*^*†*^			
< 5 days, *n* (%)	27 (27.3)	32 (29.6)	59 (28.5)
**≥ 5 days**, **n (%)**	71 (71.7)	72 (66.7)	143 (69.1)
Missing, *n* (%)	1 (1.0)	4 (3.7)	5 (2.4)
Mean (standard deviation), days	9.8 (7.7)	9.8 (13.4)	9.8 (11.0)
Median (range), days	8.0 (1, 42)	6.0 (1, 116)	7.0 (1, 116)
*Duration of prior mechanical ventilation*^*†*^			
< 5 days, *n* (%)	88 (88.9)	93 (86.1)	181 (87.4)
**≥ 5 days,** ***n* (%)**^**‡**^	11 (11.1)	12 (11.1)	23 (11.1)
Missing, *n* (%)	0	3 (2.8)	3 (1.4)
Mean (standard deviation), days	2.20 (3.08)	3.13 (8.30)	2.68 (6.33)
Median (range), days	1.18 (0.02, 20.06)	1.14 (0.04, 79.04)	1.14 (0.04, 79.04)
*PaO*_*2*_*/FiO*_*2*_			
**≤ 240 mmHg**, ***n* (%)**	75 (75.8)	79 (73.1)	154 (74.4)
> 240 mmHg,* n* (%)	23 (23.2)	27 (25.0)	50 (24.2)
Missing,* n* (%)	1 (1.0)	2 (1.9)	3 (1.4)
*Bacteremia (any pathogen)*			
**Yes, *n* (%)**	14 (14.1)	11 (10.2)	25 (12.1)
No, *n* (%)	85 (85.9)	97 (89.8)	182 (87.9)
*Number of baseline LRT pathogens*			
None confirmed, *n* (%)	24 (24.2)	24 (22.2)	48 (23.2)
Monomicrobial, *n* (%)	42 (42.4)	45 (41.7)	87 (42.0)
Polymicrobial, *n* (%)	33 (33.3)	39 (36.1)	72 (34.8)

APACHE, Acute Physiology and Chronic Health Evaluation. CPIS, Clinical Pulmonary Infection Score. C/T, ceftolozane/tazobactam. ICU, intensive care unit. ITT, intent-to-treat. NP, nosocomial pneumonia. SOFA, Sequential Organ Failure Assessment

*Antibacterial therapy active against gram-negative pathogens received in the 72 h prior to first dose of study drug

^§^Defined as adjunctive empirical therapy with amikacin, which was protocol permitted for up to 72 h at study sites where ≥ 15% of *P aeruginosa* isolates were resistant to meropenem according to the site’s most recent antibiogram

^**†**^Assessed as prior to randomization

^‡^Since some of these patients may have failed prior antibacterial therapy for ventilated NP, and because the denominator includes patients with vHABP, this number is not an exact substitute for late VABP

Baseline LRT pathogens in the vHABP subgroup were also generally comparable between treatment arms; ESBL-positive Enterobacterales and *Acinetobacter baumannii* were more frequent with ceftolozane/tazobactam and *Streptococcus* spp with meropenem (Table 2, Additional file 1: Table S2). In the ceftolozane/tazobactam arm, only 3 of 75 (4.0%) ITT participants with vHABP and confirmed baseline pathogens had a streptococcal LRT isolate. Minimum inhibitory concentration (MIC) values of gram-negative LRT pathogens to randomized study drug were MIC_50_ 2 μg/mL and MIC_90_ ≥ 256 μg/mL with ceftolozane/tazobactam and MIC_50_ < 0.064 μg/mL and MIC_90_ 32 μg/mL with meropenem. Among ITT participants with vHABP and susceptibility data available for all baseline pathogens, 37/75 (49.3%) ceftolozane/tazobactam and 26/81 (32.1%) meropenem participants had ≥ 1 baseline pathogen non-susceptible to randomized study drug. Isolates from the ceftolozane/tazobactam arm were less susceptible to non-study antibacterial agents than those from the meropenem arm (Additional file 1: Table S3). The distribution of meropenem MIC values among Enterobacterales and *P. aeruginosa* baseline LRT isolates obtained from participants with vHABP in the meropenem arm is shown in Additional file 1: Figure S2; of these, 89/99 (89.9%) isolates were fully meropenem susceptible, 1/99 intermediate susceptible (1.0%), and 9/99 (9.1%) meropenem resistant. In total, 83 of these 99 isolates (83.8%) had a meropenem MIC of ≤ 0.5 μg/m, including 73/80 (91.3%) of Enterobacterales and 10/19 (52.6%) of *P. aeruginosa* isolates. Overall baseline microbiology of LRT pathogens was generally comparable between participants with vHABP and those with VABP; the only differences (≥ 5%) were a greater incidence of *Klebsiella pneumoniae* and gram-positive pathogens in vHABP (Additional file 1: Table S4).

**Table 2:** Baseline lower respiratory tract pathogens (with a total incidence of ≥ 5%) in ASPECT-NP participants with ventilated hospital-acquired bacterial pneumonia (ITT population and mITT population)

ITT population (primary efficacy population)*			
LRT pathogen, *n* (%)	C/T (*N* = 75)	Meropenem (*N* = 84)	Total (*N* = 159)
Gram-negative	74 (98.7)	80 (95.2)	154 (96.9)
Enterobacterales	47 (62.7)	61 (72.6)	108 (67.9)
ESBL-positive Enterobacterales	27 (36.0)	25 (29.8)	52 (32.7)
* Klebsiella pneumoniae*	32 (42.7)	36 (42.9)	68 (42.8)
* Escherichia coli*	10 (13.3)	15 (17.9)	25 (15.7)
* Enterobacter cloacae*	4 (5.3)	5 (6.0)	9 (5.7)
* Proteus mirabilis*	1 (1.3)	7 (8.3)	8 (5.0)
* Pseudomonas aeruginosa*	17 (22.7)	17 (20.2)	34 (21.4)
* Acinetobacter baumannii*	19 (25.3)	17 (20.2)	36 (22.6)
* Haemophilus influenzae*	5 (6.7)	4 (4.8)	9 (5.7)
Streptococci^‡^	3 (4.0)	14 (16.7)	17 (10.7)

mITT population (secondary efficacy population)^†^			
LRT pathogen, *n* (%)	C/T (*N* = 55)	Meropenem (*N* = 71)	Total (*N* = 126)
Gram-negative	54 (98.2)	67 (94.4)	121 (96.0)
Enterobacterales	37 (67.3)	55 (77.5)	92 (73.0)
ESBL-positive Enterobacterales	17 (30.9)	23 (32.4)	40 (31.7)
* Klebsiella pneumoniae*	21 (38.2)	30 (42.3)	51 (40.5)
* Escherichia coli*	10 (18.2)	15 (21.1)	25 (19.8)
* Enterobacter cloacae*	4 (7.3)	5 (7.0)	9 (7.1)
* Proteus mirabilis*	1 (1.8)	6 (8.5)	7 (5.6)
* Pseudomonas aeruginosa*	12 (21.8)	15 (21.1)	27 (21.4)
* Acinetobacter baumannii*	5 (9.1)	6 (8.5)	11 (8.7)
* Haemophilus influenzae*	5 (9.1)	4 (5.6)	9 (7.1)
Streptococci^‡^	3 (5.5)	10 (14.1)	13 (10.3)

C/T, ceftolozane/tazobactam. ESBL, extended-spectrum β-lactamase. ITT, intent-to-treat. LRT, lower respiratory tract. mITT, microbiologic intent-to-treat. n, number of study participants with the specific pathogen. N, number of study participants in the specific treatment arm and analysis population with ≥ 1 baseline LRT

*Study participants were eligible for inclusion into the ITT population regardless of whether they had a baseline pathogen, the type of pathogen, and pathogen susceptibility

^**†**^Study participants were eligible for inclusion into the mITT population only if baseline LRT cultures yielded ≥ 1 gram-negative or streptococcal respiratory pathogen that was susceptible to ≥ 1 study drug

^‡^Since causative gram-positive LRT pathogens other than streptococci are frequently not susceptible to either study drug, microbiology data on non-streptococcal gram-positive pathogens were not captured

Baseline blood pathogens (*n* = 23 and *n* = 14 individual isolates in the ceftolozane/tazobactam and meropenem arms, respectively) were also generally comparable between treatment arms (Additional file 1: Table S5). Most frequent blood pathogens overall were staphylococci, especially *Staphylococcus epidermidis* (in 24.0% of all bacteremic participants) and *Staphylococcus hominis* (in 20.0%), as well as *A. baumannii* (in 24.0%) and *K. pneumoniae* (in 20.0%). For gram-negative blood isolates from the ceftolozane/tazobactam arm (*n* = 7), the ceftolozane/tazobactam MIC range was 0.25 to ≥ 256 μg/mL (MIC_50_ 32 μg/mL, MIC_90_ ≥ 256 μg/mL). For gram-negative blood isolates from the meropenem arm (*n* = 7), the meropenem MIC range was < 0.064 to 128 μg/mL (MIC_50_ 0.125 μg/mL, MIC_90_ 128 μg/mL).

In the vHABP subgroup, all ITT participants except for 3 in the meropenem arm received ≥ 1 dose of study drug, comprising the safety population. Treatment duration was similar between treatment arms: median (range) was 7.70 (0.7, 13.8) days in the ceftolozane/tazobactam and 7.70 (0.3, 13.8) days in the meropenem arm; 15/99 (15.2%) and 19/105 (18.1%), respectively, received ≤ 5 days of study treatment.

### Treatment outcomes

**Fig. 2:**
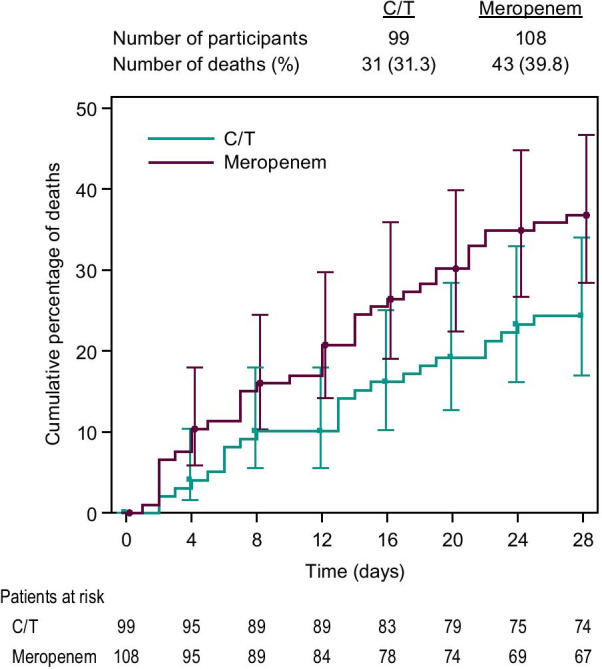
Time to death in participants with vHABP (ITT population). C/T, ceftolozane/tazobactam. ITT, intention to treat population (all randomized patients). vHABP, ventilated hospital-acquired bacterial pneumonia

Mortality rates, clinical response, and microbiologic response are shown in Table 3. Mortality was 12.8% higher in participants who received meropenem (40/108 [37.0%]) compared with those who received ceftolozane/tazobactam (24/99 [24.2%], 95% CI for difference: 0.2, 24.8), resulting in a number-needed-to-treat with ceftolozane/tazobactam (instead of meropenem) of ~ 8 patients to prevent one death. The higher mortality in the meropenem arm began to emerge on day 2 and continued to diverge through day 28 (Fig. 2). When evaluating 28-day ACM in participants with vHABP by various clinically relevant baseline characteristics, most comparisons indicated comparable mortality between treatment groups. The exceptions (with observed lower mortality in the ceftolozane/tazobactam arm) included: prolonged prior mechanical ventilation, prolonged prior hospitalization, and SOFA score > 7 (Additional file 1: Table S6). In participants receiving protocol-permitted adjunctive gram-negative empirical therapy (almost exclusively amikacin), 28-day ACM rates were 10/35 (28.6%) with ceftolozane/tazobactam and 14/34 (41.2%) with meropenem (95% CI for difference: − 9.6, 33.2). In participants with *P. aeruginosa* at baseline, mortality was 2/17 (11.8%) and 5/17 (29.4%), respectively (95% CI for difference: − 10.1, 42.8). Safety, as measured by different types of adverse event rates, was generally comparable between treatment arms (Table 4). However, participants in the meropenem arm had a higher rate of adverse events leading to study drug discontinuation (17.1% vs 8.1% with ceftolozane/tazobactam).

**Table 3:** Primary and secondary efficacy outcomes in ASPECT-NP participants with ventilated hospital-acquired bacterial pneumonia by treatment arm

Endpoint	C/T *n/N* (%)	Meropenem *n/N* (%)	% Difference (95% CI)^**^
28-day all-cause mortality (ITT)*	24/99 (24.2%)	40/108 (37.0%)	12.8% (0.2, 24.8)
All LRT pathogens susceptible to randomized study drug^‡^	7/38 (18.4%)	20/55 (36.4%)	17.9% (− 0.9, 34.0)
≥ 1 LRT pathogen non-susceptible to randomized study drug^‡^	10/37 (27.0%)	11/26 (42.3%)	15.3% (− 7.9, 37.3)
28-day all-cause mortality (mITT)^*^	10/55 (18.2%)	26/71 (36.6%)	18.4% (2.5, 32.5)
Monomicrobial	5/33 (15.2%)	16/40 (40.0%)	24.8% (4.0, 42.4)
Polymicrobial	5/22 (22.7%)	10/31 (32.3%)	9.5% (− 15.3, 31.2)
Clinical cure at TOC (ITT)*	50/99 (50.5%)	48/108 (44.4%)	6.1% (− 7.4, 19.3)
Clinical cure at TOC (CE)^§^	34/59 (57.6%)	32/49 (65.3%)	− 7.7% (− 25.0, 10.6)
Microbiologic eradication at TOC (mITT)*^,¶^	43/55 (78.2%)	44/71 (62.0%)	16.2% (− 0.1, 30.8)
Microbiologic eradication at TOC (ME)*^,¶^	15/21 (71.4%)	16/25 (64.0%)	7.4% (− 19.1, 31.9)

CE, clinically evaluable. CI, confidence interval. C/T, ceftolozane/tazobactam. ITT, intent-to-treat. ME, microbiologically evaluable. mITT, microbiological intent-to-treat. TOC, test-of-cure

*Patients with missing/indeterminate data are reported as deceased or as failures, depending on the endpoint

^‡^Not all ITT patients had confirmed baseline pathogens and susceptibility data available

^§^Data reported as observed, i.e., patients with missing/indeterminate responses excluded from analysis

^¶^Per-patient microbiologic eradication

**Unstratified Newcombe CIs; positive differences are in favor of ceftolozane/tazobactam, negative differences are in favor of meropenem

**Table 4:** Adverse event summary in ASPECT-NP participants with ventilated hospital-acquired bacterial pneumonia by treatment arm

Adverse event category,* n* (%)	C/T (*N* = 99)	Meropenem (*N* = 105)	Total (*N* = 204)
Number of patients with ≥ 1 TEAE	88 (88.9)	92 (87.6)	180 (88.2)
Number of patients with ≥ 1 TEAE by maximum severity*			
Mild	15 (15.2)	14 (13.3)	29 (14.2)
Moderate	30 (30.3)	28 (26.7)	58 (28.4)
Severe	43 (43.4)	50 (47.6)	93 (45.6)
Number of patients with ≥ 1 DRAE^‡^	13 (13.1)	11 (10.5)	24 (11.8)
Number of patients with ≥ 1 serious TEAE	49 (49.5)	48 (45.7)	97 (47.5)
Number of patients with ≥ 1 serious DRAE^‡^	3 (3.0)	1 (1.0)	4 (2.0)
Number of patients with ≥ 1 TEAE leading to study drug discontinuation	8 (8.1)	18 (17.1)	26 (12.7)
Number of patients with ≥ 1 DRAE leading to study drug discontinuation^‡^	2 (2.0)	1 (1.0)	3 (1.5)
Number of patients with ≥ 1 TEAE resulting in death	31 (31.3)	41 (39.0)	72 (35.3)
Number of patients with ≥ 1 DRAE resulting in death^‡^	0	0	0

Adverse events were coded using MedDRA version 17.0. For each category, patients counted only once, even if they experienced multiple events in the category

AE, adverse event. C/T, ceftolozane/tazobactam. DRAE, drug-related adverse event. *n*, number of patients in specific category. *N*, number of patients in safety population. TEAE, treatment-emergent adverse event (defined as any AE starting on/after the first study drug administration)

*If patients have multiple events, they are counted only once with the maximum (highest) severity rating. The orders of severity from worst case to best case are severe, moderate, and mild

^‡^If a patient had multiple events and ≥ 1 was deemed related to study drug, then that patient was included into the count

### Multivariable analysis

As detailed above, forward selection logistic regression (informed by results from a random forest analysis) was used to select the explanatory variables for inclusion into the final regression model. The 200 random forest model runs had a median (interquartile range) OOB error rate of 36.7% (33.7–38.2%). The ranking of the 16 preselected variables in order of most to least impact on 28-day ACM is shown in Additional file 1: Figure S1. Forward selection logistic regression was then used to determine which of the 16 variables (treated as continuous whenever possible) had the greatest impact on mortality and should be included into the final regression model; the order in which variables were entered into the forward regression model was identical to the ranking established by the random forest analysis. In forward regression, the area under the ROC increased to approximately 0.70 when the 4 top-ranking variables (i.e., concomitant vasopressor use, baseline age, baseline bacteremia, and baseline PaO_2_/FiO_2_) were entered into the model; no additional gain in ROC was obtained when additional variables were included. The initial, variable selection step of this multivariable analysis therefore showed that the four most important factors influencing 28-day ACM in the vHABP subgroup were: concomitant vasopressor use (categorical variable), baseline age (continuous variable), baseline bacteremia (categorical variable), and baseline PaO_2_/FiO_2_ (continuous variable) (Additional file 1: Figure S1).

The four factors, in combination with treatment (i.e., ceftolozane/tazobactam vs meropenem), were further evaluated in a backward elimination logistic main effects regression model. Treatment, bacteremia, and vasopressor use remained significant (*p* < 0.05) in the final regression model, while age and baseline PaO_2_/FiO_2_ were removed from the model due to lack of significant impact on 28-day all-cause mortality. Since this was a main effects model, no interaction terms were included. The final model had an area under the ROC curve of 0.74, indicating that successful classification of mortality is achieved with this fitted model. ORs for death by day 28 (Table 5) were 5.4 for vasopressor use (adjusting for treatment and bacteremia) and 2.7 for bacteremia (adjusting for treatment and vasopressor use). Treatment was also significantly associated with mortality (adjusting for vasopressor use and bacteremia): the OR with meropenem treatment (vs ceftolozane/tazobactam) was 2.3 (95% CI 1.2, 4.5). Results of the sensitivity analysis were fully consistent with those of the main multivariable analysis, with treatment, vasopressor use, and bacteremia as the only factors significantly associated with mortality (Additional file 1: Table S7).

**Table 5:** Odds ratio estimates (and confidence intervals) for risk of death due to any cause by day 28 associated with the significant factors included into the final logistic regression model, each adjusted for both of the other factors

Patient characteristic	Odds ratio for 28-day ACM (95% CI)
Baseline bacteremia with any pathogen (vs no bacteremia)^*^	2.7 (1.1, 7.1)
Concomitant vasopressor use (vs no vasopressor use)^‡^	5.4 (2.6, 11.0)
Meropenem treatment (vs ceftolozane/tazobactam treatment)^§^	2.3 (1.2, 4.5)

ACM, all-cause mortality. CI, confidence interval

^*^Adjusting for vasopressor use and treatment

^‡^Adjusting for bacteremia and treatment

^§^Adjusting for bacteremia and vasopressor use

## Discussion

The ASPECT-NP randomized, controlled, phase 3 trial previously demonstrated noninferiority of ceftolozane/tazobactam to meropenem for treating mechanically ventilated participants with HABP or VABP in both the primary endpoint of 28-day ACM and the key secondary endpoint of clinical cure at the test-of-cure visit, respectively [14]. A key subgroup of the trial were participants with vHABP, the type of nosocomial pneumonia with the highest mortality. In this subgroup, ceftolozane/tazobactam resulted in lower 28-day ACM than meropenem in both ITT and mITT populations. Multivariable analysis incorporating baseline factors, treatment factors, and causative microbiology demonstrated that two factors associated with disease severity (i.e., vasopressor use and bacteremia) were most strongly associated with higher mortality risk, independent of treatment arm. When controlling for these factors, treatment of vHABP with meropenem was still associated with more than twice the risk of death compared to treatment with ceftolozane/tazobactam. While this limited, retrospective analysis cannot be relied upon to determine the true magnitude of the increased mortality risk, our results are still clinically relevant, because treatment regimen was the only potentially modifiable factor contributing to lower mortality. This supports the hypothesis that ceftolozane/tazobactam may confer a survival advantage over meropenem in the high-risk subpopulation of participants with gram-negative vHABP, but this requires confirmation in an adequately powered trial.

Previous randomized controlled trials in nosocomial pneumonia have consistently demonstrated higher mortality in patients with vHABP than those with VABP [1]. In line with this trend, ASPECT-NP participants in the meropenem arm had higher mortality when treated for vHABP (i.e., 37%) than for VABP (20%). In the ceftolozane/tazobactam arm, however, 28-day ACM rates were identical (i.e., 24%) in both the vHABP and VABP subgroups, indicating that the mortality difference between ceftolozane/tazobactam and meropenem in participants with vHABP was driven by a lower-than-expected mortality in ceftolozane/tazobactam-treated participants and not higher-than-expected mortality in those receiving meropenem. (A potential survival difference between 2 antibacterial agents is most likely to become apparent in those patient populations at the highest mortality risk.) It is important to note that all-cause mortality rather than pneumonia-attributable mortality was evaluated in this trial. Similar to participants in other HABP/VABP studies, those enrolled in ASPECT-NP were often critically ill due to conditions (resulting in their initial hospitalization) other than pneumonia or had significant comorbidities. These confounding factors make it difficult to distinguish whether deaths occurring in this study were directly related to the current pneumonia episode or influenced by participants’ underlying factors. Although the randomized study design should largely have controlled for confounders, it is still possible that the mortality differences were ultimately driven by a lower than expected rate of non-pneumonia–related mortality in the ceftolozane/tazobactam arm.

A mortality difference in favor of ceftolozane/tazobactam among participants with vHABP was also observed in the mITT population, a subset of the primary efficacy ITT population that excluded participants with only resistant pathogens or without any confirmed LRT pathogens; the mITT population was thus less at risk of potential bias than the ITT. Just as in the ITT, the 95% CI for the mortality difference in the mITT population also excluded zero and thus provides additional support to the hypothesis of a potential survival advantage with ceftolozane/tazobactam in vHABP. For the key secondary endpoint of clinical cure, the 95% CI for the difference between treatment arms in ITT participants with vHABP did not exclude zero, although a trend toward slightly higher cure rates with ceftolozane/tazobactam vs meropenem was seen. Similarly, a trend toward higher microbiologic eradication rates in the ceftolozane/tazobactam arm was found. In part, these lesser differences may reflect lower event rates and the smaller sample size of these subgroups. Finally, the mortality difference between treatment arms started to become apparent around day 2 of treatment and persisted throughout the treatment period—this timing aligns with the expected timepoint of antibiotic treatment failure [20, 21]. All of these observations support the hypothesis that the observed mortality difference was due to differences in the antibacterial treatments administered, rather than due to other underlying factors.

Baseline characteristics and causative pathogens were as expected in this patient population [22]. When analyzing baseline LRT pathogen susceptibility to randomized study drug in the vHABP subpopulation, three observations would actually lead us to expect a bias in favor of meropenem: first, baseline pathogens in the meropenem arm had lower MIC values to meropenem than conversely those in the ceftolozane/tazobactam arm had to ceftolozane/tazobactam arm; second, more pathogens in the ceftolozane/tazobactam arm were non-susceptible to ceftolozane/tazobactam than vice versa in the meropenem arm; and third, almost 20% fewer meropenem (32%) than ceftolozane/tazobactam participants (49%) had ≥ 1 LRT baseline pathogen non-susceptible to randomized study drug. It should be noted that in ASPECT-NP, meropenem was administered at a dose of 1 g q8h via 1-h infusion, according to clinical guidelines in place when the study commenced [14]. However, optimized meropenem dosing by administering high-dose (e.g., 2 g q8h), extended (e.g., 3 h) infusions is increasingly being recommended in critically ill patients with nosocomial pneumonia [23–27], in whom meropenem epithelial lining fluid (ELF) penetration can be highly variable [28]. This dosing strategy may particularly improve outcomes in pathogens with meropenem MIC values ≥ 1 μg/mL, as well as overcome inadequate meropenem levels caused by presence of renal hyperclearance; in patients with normal renal function, on the other hand, standard meropenem dosing may be sufficient [29]. Extended infusion regimens may also be beneficial for HABP/VABP due to *P. aeruginosa* or *Acinetobacter* spp [30]. However, the 1 g q8h meropenem dosing regimen is unlikely to have impacted mortality results in our analysis, since among participants with vHABP in the meropenem arm, < 5% had ARC and about 85% of relevant baseline LRT isolates had low meropenem MIC values of ≤ 0.5 μg/mL. This indicates that only very few of these trial participants might potentially have benefited from high-dose, extended meropenem infusions. However, given the significant inter-patient variability in meropenem ELF penetration [28], it is unknown to what extent optimized dosing might have improved outcomes in the meropenem arm.

In order to determine if treatment selection (i.e., ceftolozane/tazobactam vs meropenem) differentially impacted ACM even when adjusting for other clinically relevant factors, we conducted a rigorous multivariable analysis. Variables with a significant impact on 28-day ACM in the final model were study treatment, vasopressor use, and bacteremia. Participants with vHABP had a 5 times greater risk of death if requiring vasopressors and a 2 times greater risk if bacteremic at baseline. Adjusting for vasopressor use and bacteremia, participants were over twice as likely to die by 28 days if given meropenem instead of ceftolozane/tazobactam. These explanatory variables match expectations from existing data. Treatment selection (i.e., administration of an antibacterial agent, at a sufficient dose, that the causative pathogen is susceptible to) is well known to be one of the most important prognostic factors in patients with HABP/VABP [31–33]. Similarly, septic shock (indicated by vasopressor use) and concurrent bacteremia have frequently been reported as adversely affecting treatment outcomes in nosocomial pneumonia [34, 35].

Conversely, several variables previously reported as having prognostic significance in HABP/VABP [34] were not found influential in our model, such as SOFA score and oxygenation levels. Of note, several of the independent variables preselected for potential evaluation in the regression model are known or expected to have a high degree of collinearity: for example, SOFA score is colinear with both vasopressor use and PaO_2_/FiO_2_, because both of these are components of SOFA score calculation. Since multicollinearity negatively affects model fit and interpretation, selection of the most impactful and explanatory subset of independent variables using appropriate variable selection techniques was particularly important. For example, univariate analysis cannot account for collinearity (since this type of analysis only evaluates the relationship of a single independent variable) and would therefore not have been suitable for evaluating the question at hand. We used the random forest algorithm to determine the order in which independent, exploratory variables were entered into the regression model through standard forward stepwise selection. The random forest algorithm, a supervised learning method, has several advantages over alternatives, including a more powerful and robust predictive performance and the ability to address nonlinear relationships and complex interactions between the included variables [18, 19, 36, 37]. One disadvantage of this method, however, is decreased interpretability compared to other, traditional approaches. For this reason, a traditional multivariable logistic regression analysis was performed, results of which were fully consistent with the main approach. Caution should be used when interpreting the results of the multivariable analyses, especially the values of the ORs. While the model confirmed that vasopressor use, bacteremia, and treatment assignment significantly influenced 28-day all-cause mortality, the actual magnitude of influence cannot be firmly concluded from our data. For instance, bacteremia was found to be a significant factor, but very few trial participants actually presented with baseline bacteremia.

The ASPECT-NP trial design itself was also associated with several notable strengths, such as the fact that this study enrolled critically ill participants representative of the target population in terms of baseline clinical and demographic characteristics, similar to real-world patients with mechanically ventilated nosocomial pneumonia [14]. In addition, randomization was stratified by vHABP vs VABP [14], lending additional robustness to the subgroup analyses described here. On the other hand, our exploratory analyses are limited by their retrospective nature, the smaller sample size of the vHABP subgroup overall, and the low frequency of some of the key baseline factors (in particular bacteremia). Finally, we cannot exclude the possibility that imbalances in non-pneumonia–related deaths between treatment arms or unknown imbalances in baseline characteristics were the driver behind the observed differences. While unknown baseline imbalances could be an alternative explanation for the rapid separation of survival curves by Day 2, this is less likely, considering that the stratified randomization process makes such imbalances improbable. Adequately powered prospective studies are therefore required to confirm the potential survival advantage of ceftolozane/tazobactam over meropenem in vHABP.

## Conclusions

In summary, the previously reported difference in 28-day all-cause mortality in the vHABP subgroup did not appear to be due to clinically meaningful differences in patient characteristics, causative pathogens, or antibacterial susceptibility. A multivariable analysis provided further evidence for a mortality difference in participants with vHABP receiving meropenem instead of ceftolozane/tazobactam when adjusting for other factors that significantly impacted mortality in this patient population. While this retrospective analysis is unable to account for all possible confounding factors, our data suggest a potential survival advantage with ceftolozane/tazobactam in this high-risk, difficult-to-treat subpopulation that needs to be confirmed in adequately powered prospective studies.

## Supplementary Information

**Additional file 1: Table S1**. Baseline demographics and clinical characteristics in ASPECT-NP participants with ventilated hospital-acquired vs those with ventilator-associated bacterial pneumonia (ITT population, both treatment arms combined). **Table S2**. Baseline microbiologic assessment in ASPECT-NP participants with ventilated hospital-acquired bacterial pneumonia (ITT population). **Table S3**. Susceptibility of baseline* Pseudomonas aeruginosa* and Enterobacterales to key antibacterial agents, by treatment arm, in ASPECT-NP participants with ventilated hospital-acquired bacterial pneumonia (ITT population). **Table S4**. Baseline microbiologic assessment in ASPECT-NP participants with ventilated hospital-acquired vs those with ventilator-associated bacterial pneumonia (ITT population, both treatment arms combined).** Table S5**. Baseline bacterial pathogen isolates from blood cultures in the ventilated HABP subgroup, by treatment arm (ITT population). **Table S6**. 28-day all-cause mortality in participants with vHABP, by clinical characteristics evaluated in the multivariable analysis (ITT population).** Table S7**. Odds ratio estimates (and confidence intervals) for risk of death due to any cause by day 28 associated with the significant factors in the sensitivity logistic regression model. **Figure S1**. Methodology and general results of the multivariable analysis. **Figure S2**. Frequency distribution of meropenem MIC values for (A) Enterobacterales (N = 80 isolates) and (B)* Pseudomonas aeruginosa* (N = 19) isolates obtained from participants with vHABP of the meropenem arm.

## Data Availability

Merck Sharp & Dohme Corp., a subsidiary of Merck & Co., Inc., Kenilworth, NJ, USA’s data sharing policy, including restrictions, is available at http://engagezone.msd.com/ds_documentation.php. Requests for access to the clinical study data can be submitted through the EngageZone site or via email to dataaccess@merck.com.

## Abbreviations

ACM: All-cause mortality
APACHE: Acute Physiology and Chronic Health Evaluation
ARC: Augmented renal clearance
CE: Clinically evaluable
CFU: Colony-forming unit
CI: Confidence interval
CLSI: Clinical and Laboratory Standards Institute
CPIS: Clinical Pulmonary Infection Score
CrCL: Creatinine clearance
C/T: Ceftolozane/tazobactam
DRAE: Drug-related adverse event
ELF: Epithelial lining fluid
EOT: End-of-therapy
ESBL: Extended-spectrum β-lactamases
HABP: Hospital-acquired bacterial pneumonia
ICU: Intensive care unit
ITT: Intent-to-treat
LRT: Lower respiratory tract
LFU: Late follow-up
MBL: Metallo-β-lactamases
ME: Microbiologically evaluable
MedDRA: Medical Dictionary for Regulatory Activities
MIC: Minimum inhibitory concentration
mITT: Microbiologic intent-to-treat
NC: North Carolina
OOB: Out of bag
OR: Odds ratio
PaO_2_/FiO_2_: Ratio of arterial oxygen partial pressure to fractional inspired oxygen
q8h: Every 8 h
ROC: Receiver operating characteristic
SAS: Statistical Analysis Software
SOFA: Sequential Organ Failure Assessment
TEAE: Treatment-emergent adverse event
TOC: Test-of-cure
VABP: Ventilator-associated bacterial pneumonia
vHABP: Ventilated hospital-acquired bacterial pneumonia
vNP: Ventilated nosocomial pneumonia
