# Supplementary material for: Ceftolozane/tazobactam versus meropenem in patients with ventilated hospital-acquired bacterial pneumonia: subset analysis of the ASPECT-NP randomized, controlled phase 3 trial
Source: Crit Care. 2021 Aug 11;25:290. doi: 10.1186/s13054-021-03694-3 (PMC8356211; doi:10.1186/s13054-021-03694-3)

**Online supplementary appendix to:**

**Ceftolozane/tazobactam vs meropenem in patients with ventilated hospital-acquired pneumonia – subset analysis of the ASPECT-NP randomized, controlled phase 3 trial**

Table of Contents

[Table S1: Baseline demographics and clinical characteristics in ASPECT-NP participants with ventilated hospital-acquired vs those with ventilator-associated bacterial pneumonia (ITT population, both treatment arms combined). 3](#_Toc76725342)

[Table S2: Baseline microbiologic assessment in ASPECT-NP participants with ventilated hospital-acquired bacterial pneumonia (ITT population). 7](#_Toc76725343)

[Table S3: Susceptibility of baseline *Pseudomonas* *aeruginosa* and Enterobacterales to key antibacterial agents, by treatment arm, in ASPECT-NP participants with ventilated hospital-acquired bacterial pneumonia (ITT population). 11](#_Toc76725344)

[Table S4: Baseline microbiologic assessment in ASPECT-NP participants with ventilated hospital-acquired vs those with ventilator-associated bacterial pneumonia (ITT population, both treatment arms combined). 13](#_Toc76725345)

[Table S5: Baseline bacterial pathogen isolates from blood cultures in the ventilated HABP subgroup, by treatment arm (ITT population). 17](#_Toc76725346)

[Table S6: 28-day all-cause mortality in participants with vHABP, by clinical characteristics evaluated in the multivariable analysis (ITT population). 19](#_Toc76725347)

[Table S7: Odds ratio estimates (and confidence intervals) for risk of death due to any cause by day 28 associated with the significant factors in the sensitivity logistic regression model. 21](#_Toc76725348)

[Figure S1. Methodology and general results of the multivariable analysis. 22](#_Toc76725349)

[Figure S2. Frequency distribution of meropenem MIC values for (A) Enterobacterales (N=80 isolates) and (B) *Pseudomonas aeruginosa* (N=19) isolates obtained from participants with vHABP of the meropenem arm. Breakpoints indicated reflect current (2021) CLSI susceptibility breakpoints for meropenem. 23](#_Toc76725350)

# Table S1: Baseline demographics and clinical characteristics in ASPECT-NP participants with ventilated hospital-acquired vs those with ventilator-associated bacterial pneumonia (ITT population, both treatment arms combined).

Known prognostic factors for worse clinical outcomes are underlined.

|  | **Ventilated HABP**  **(N=207)** | **VABP**  **(N=519)** |
| --- | --- | --- |
| **Sex** |  |  |
| Male, n (%) | 146 (70.5) | 371 (71.5) |
| Female, n (%) | 61 (29.5) | 148 (28.5) |
| **Age, years** |  |  |
| <65, n (%) | 99 (47.8) | 307 (59.2) |
| ≥65, n (%) | 108 (52.2) | 212 (40.8) |
| Mean (standard deviation) | 64.3 (14.9) | 58.3 (17.4) |
| Median (range) | 65.0 (18, 98) | 61.0 (18, 90) |
| **Weight, kg** |  |  |
| Median (range) | 78.0 (34.0, 151.0) | 80.0 (41.0, 225.0) |
| **Body-mass index, kg** |  |  |
| Median (range) | 26.1 (15.1, 49.3) | 26.9 (15.6, 67.2) |
| **Creatinine clearance (mL/min)** |  |  |
| ≥150 (augmented renal clearance), n (%) | 14 (6.8) | 117 (22.5) |
| ≥80, n (%) | 92 (44.4) | 371 (71.5) |
| <80 to >50, n (%) | 60 (29.0) | 99 (19.1) |
| ≤50 to ≥30, n (%) | 27 (13.0) | 34 (6.6) |
| <30 to ≥15, n (%) | 25 (12.1) | 13 (2.5) |
| <15 (end-stage renal disease), n (%) | 0 | 1 (0.2) |
| Missing, n (%) | 3 (1.4) | 1 (0.2) |
| **In the ICU** |  |  |
| Yes, n (%) | 169 (81.6) | 499 (96.1) |
| No, n (%) | 38 (18.4) | 20 (3.9) |
| **APACHE II score** |  |  |
| ≤14, n (%) | 59 (28.5) | 123 (23.7) |
| 15-19, n (%) | 76 (36.7) | 226 (43.5) |
| ≥20, n (%) | 71 (34.3) | 168 (32.4) |
| Missing, n (%) | 1 (0.5) | 2 (0.4) |
| Mean (standard deviation) | 18.0 (6.0) | 17.3 (5.2) |
| Median (range) | 17.0 (4, 38) | 17.0 (2, 39) |
| **SOFA score** |  |  |
| ≤7, n (%) | 122 (58.9) | 376 (72.4) |
| >7, n (%) | 83 (40.1) | 143 (27.6) |
| Missing | 2 (1.0) | 0 |
| **Prior non-study gram-negative therapy^*^** |  |  |
| Yes, n (%) | 185 (89.4) | 456 (87.9) |
| No, n (%) | 22 (10.6) | 63 (12.1) |
| **CPIS** |  |  |
| ≤6, n (%) | 16 (7.7) | 41 (7.9) |
| 7, n (%) | 19 (9.2) | 45 (8.7) |
| 8, n (%) | 26 (12.6) | 61 (11.8) |
| >8, n (%) | 145 (70.0) | 372 (71.7) |
| Missing | 1 (0.5) | 0 |
| **Duration of prior hospitalization^†^** |  |  |
| <5 days, n (%) | 59 (28.5) | 102 (19.7) |
| ≥5 days, n (%) | 143 (69.1) | 414 (79.8) |
| Missing, n (%) | 5 (2.4) | 3 (0.6) |
| Mean (standard deviation), days | 9.8 (11.0) | 10.6 (20.0) |
| Median (range), days | 7.0 (1, 116) | 8.0 (1, 418) |
| **Duration of prior mechanical ventilation^†^** |  |  |
| <5 days, n (%) | 181 (87.4) | 181 (34.9) |
| ≥5 days, n (%)^‡^ | 23 (11.1) | 335 (64.5) |
| Missing, n (%) | 3 (1.4) | 3 (0.6) |
| Mean (standard deviation), days | 2.68 (6.33) | 11.00 (43.50) |
| Median (range), days | 1.14 (0.02, 79.04) | 6.36 (2.00, 767.40) |
| **Failed prior antibacterial therapy for NP** |  |  |
| Yes, n (%) | 37 (17.9) | 56 (10.8) |
| No, n (%) | 169 (81.6) | 463 (89.2) |
| Missing | 1 (0.5) | 0 |
| **Bacteremia (any pathogen)** |  |  |
| Yes, n (%) | 25 (12.1) | 80 (15.4) |
| No, n (%) | 182 (87.9) | 439 (84.6) |
| **Concomitant vasopressor use^§^** |  |  |
| Yes, n (%) | 110 (53.1) | 209 (40.3) |
| No, n (%) | 97 (46.9) | 488 (59.7) |

APACHE, Acute Physiology and Chronic Health Evaluation. CPIS, Clinical Pulmonary Infection Score. C/T, ceftolozane/tazobactam. ICU, intensive care unit. ITT, intent-to-treat. NP, ventilated nosocomial pneumonia. SOFA, Sequential Organ Failure Assessment.

**^*^**Antibacterial therapy active against gram-negative pathogens received in the 72 h prior to first dose of study drug. **^†^**Assessed as prior to randomisation. ^‡^Since some of these patients may have failed prior antibacterial therapy for ventilated NP, and because the denominator includes patients with ventilated HABP, this number is not an exact substitute for late VABP. §Not a baseline factor; concomitant vasopressor use was defined as adrenergic and/or dopaminergic agents administered anytime from first dose of study drug to before the late follow-up visit.

# Table S2: Baseline microbiologic assessment in ASPECT-NP participants with ventilated hospital-acquired bacterial pneumonia (ITT population).

Results are shaded for the treatment arm in which the corresponding pathogen/category was ≥5% more frequent at baseline.

1. Baseline lower respiratory tract pathogen (of any incidence)

| **Baseline LRT pathogen(s), n (%) *** | **C/T (N=75)** | **Meropenem (N=84)** | **Total (N=159)** |
| --- | --- | --- | --- |
| **Gram-negative pathogens** | **74 (98.7)** | **80 (95.2)** | **154 (96.9)** |
| *Pseudomonas aeruginosa* | 17 (22.7) | 17 (20.2) | 34 (21.4) |
| AmpC-overexpressing *Pseudomonas aeruginosa* | 3 (4.0) | 1 (1.2) | 4 (2.5) |
| Enterobacterales | 47 (62.7) | 61 (72.6) | 108 (67.9) |
| ESBL+ Enterobacterales | 27 (36.0) | 25 (29.8) | 52 (32.7) |
| *Citrobacter freundii* | 0 | 1 (1.2) | 1 (0.6) |
| *Citrobacter koseri* | 2 (2.7) | 3 (3.6) | 5 (3.1) |
| *Enterobacter cloacae* | 4 (5.3) | 5 (6.0) | 9 (5.7) |
| ESBL+ *Enterobacter cloacae* | 0 | 1 (1.2) | 1 (0.6) |
| *Escherichia coli* | 10 (13.3) | 15 (17.9) | 25 (15.7) |
| ESBL+ *Escherichia coli* | 4 (5.3) | 3 (3.6) | 7 (4.4) |
| *Escherichia hermannii* | 0 | 1 (1.2) | 1 (0.6) |
| ESBL+ *Escherichia hermannii* | 0 | 1 (1.2) | 1 (0.6) |
| *Klebsiella (Enterobacter) aerogenes* | 3 (4.0) | 3 (3.6) | 6 (3.8) |
| *Klebsiella oxytoca* | 1 (1.3) | 4 (4.8) | 5 (3.1) |
| ESBL+ *Klebsiella oxytoca* | 0 | 2 (2.4) | 2 (1.3) |
| *Klebsiella pneumoniae* | 32 (42.7) | 36 (42.9) | 68 (42.8) |
| ESBL+ *Klebsiella pneumoniae* | 23 (30.7) | 20 (23.8) | 43 (27.0) |
| *Morganella morganii* | 1 (1.3) | 1 (1.2) | 2 (1.3) |
| *Pantoea agglomerans* | 1 (1.3) | 0 | 1 (0.6) |
| *Proteus mirabilis* | 1 (1.3) | 7 (8.3) | 8 (5.0) |
| ESBL+ *Proteus mirabilis* | 1 (1.3) | 3 (3.6) | 4 (2.5) |
| *Proteus vulgaris* | 0 | 1 (1.2) | 1 (0.6) |
| *Serratia marcescens* | 3 (4.0) | 5 (6.0) | 8 (5.0) |
| ESBL+ *Serratia marcescens* | 1 (1.3) | 1 (1.2) | 2 (1.3) |
| *Achromobacter xylosoxidans* | 1 (1.3) | 2 (2.4) | 3 (1.9) |
| *Acinetobacter baumannii* | 19 (25.3) | 17 (20.2) | 36 (22.6) |
| *Acinetobacter calcoaceticus* | 1 (1.3) | 0 | 1 (0.6) |
| *Acinetobacter lwoffii* | 0 | 2 (2.4) | 2 (1.3) |
| *Acinetobacter* sp | 0 | 1 (1.2) | 1 (0.6) |
| *Aeromonas hydrophila* | 0 | 1 (1.2) | 1 (0.6) |
| *Burkholderia cepacia* | 2 (2.7) | 0 | 2 (1.3) |
| *Burkholderia vesicularis* | 1 (1.3) | 0 | 1 (0.6) |
| *Delftia acidovorans* | 0 | 1 (1.2) | 1 (0.6) |
| *Haemophilus influenzae* | 5 (6.7) | 4 (4.8) | 9 (5.7) |
| *Haemophilus* sp | 1 (1.3) | 0 | 1 (0.6) |
| *Neisseria meningitidis* | 1 (1.3) | 0 | 1 (0.6) |
| *Stenotrophomonas maltophilia* | 2 (2.7) | 2 (2.4) | 4 (2.5) |
| **Gram-positive pathogens** | **3 (4.0)** | **14 (16.7)** | **17 (10.7)** |
| *Streptococcus agalactiae* | 0 | 1 (1.2) | 1 (0.6) |
| *Streptococcus mitis* | 0 | 1 (1.2) | 1 (0.6) |
| *Streptococcus pneumoniae* | 1 (1.3) | 6 (7.1) | 7 (4.4) |
| *Streptococcus* sp, alpha hemolytic | 0 | 3 (3.6) | 3 (1.9) |
| *Streptococcus* sp, beta hemolytic | 1 (1.3) | 1 (1.2) | 2 (1.3) |
| *Streptococcu*s sp, non-hemolytic | 0 | 2 (2.4) | 2 (1.3) |
| *Streptococcus viridans* | 1 (1.3) | 1 (1.2) | 2 (1.3) |

^*^Participants with more than 1 pathogen isolated at baseline are counted only once within each pathogen category.

1. Number of different lower respiratory tract pathogens identified at baseline

| **Number of baseline LRT pathogens, n (%)** | **C/T (N=75)** | **Meropenem (N=84)** | **Total (N=159)** |
| --- | --- | --- | --- |
| 1 | 42 (56.0) | 45 (53.6) | 87 (54.7) |
| 2 | 30 (40.0) | 21 (25.0) | 51 (32.1) |
| 3 | 3 (4.0) | 15 (17.9) | 18 (11.3) |
| >3 | 0 | 3 (3.6) | 3 (1.9) |

1. Method used for lower respiratory tract specimen collection

| **Specimen collection method, n (%)** | **C/T (N=75)** | **Meropenem (N=84)** | **Total (N=159)** |
| --- | --- | --- | --- |
| Endotracheal aspirate | 40 (53.3) | 45 (53.6) | 85 (53.5) |
| Bronchoalveolar lavage | 16 (21.3) | 18 (21.4) | 34 (21.4) |
| Mini-bronchoalveolar lavage | 17 (22.7) | 20 (23.8) | 37 (23.3) |
| Protected brush specimen | 2 (2.7) | 1 (1.2) | 3 (1.9) |

# Table S3: Susceptibility of baseline *Pseudomonas* *aeruginosa* and Enterobacterales to key antibacterial agents, by treatment arm, in ASPECT-NP participants with ventilated hospital-acquired bacterial pneumonia (ITT population).

|  | **C/T** | | **Meropenem** | | **Amikacin** | | **Cefepime** | |
| --- | --- | --- | --- | --- | --- | --- | --- | --- |
|  | **C/T**  **arm** | **Meropenem arm** | **C/T**  **arm** | **Meropenem arm** | **C/T**  **arm** | **Meropenem arm** | **C/T**  **arm** | **Meropenem arm** |
|  | **n/N1 (%)** | **n/N1 (%)** | **n/N1 (%)** | **n/N1 (%)** | **n/N1 (%)** | **n/N1 (%)** | **n/N1 (%)** | **n/N1 (%)** |
| **Overall** | **49/75 (65.3)** | **77/96 (80.2)** | **55/75 (73.3)** | **87/97 (89.7)** | **56/69 (81.2)** | **73/89 (82.0)** | **31/69 (44.9)** | **58/89 (65.2)** |
| ***Pseudomonas aeruginosa*** | **12/17 (70.6)** | **15/17 (88.2)** | **9/17 (52.9)** | **13/17 (76.5)** | **10/17 (58.8)** | **14/17 (82.4)** | **9/17 (52.9)** | **15/17 (88.2)** |
| **Enterobacterales** | **37/58 (63.8)** | **62/79 (78.5)** | **46/58 (79.3)** | **74/80 (92.5)** | **46/52 (88.5)** | **59/72 (81.9)** | **22/52 (42.3)** | **43/72 (59.7)** |
| ESBL+ Enterobacterales | 10/29 (34.5) | 17/31 (54.8) | 18/29 (62.1) | 26/30 (86.7) | 23/29 (79.3) | 21/31 (67.7) | 0/29 (0.0) | 5/31 (16.1) |
| *Citrobacter freundii* | 0/0 | 0/1 (0.0) | 0/0 | 1/1 (100.0) | 0/0 | 0/1 (0.0) | 0/0 | 1/1 (100.0) |
| *Citrobacter koseri* | 2/2 (100.0) | 3/3 (100.0) | 2/2 (100.0) | 3/3 (100.0) | 2/2 (100.0) | 1/1 (100.0) | 2/2 (100.0) | 1/1 (100.0) |
| *Enterobacter cloacae* | 3/4 (75.0) | 4/5 (80.0) | 4/4 (100.0) | 5/5 (100.0) | 4/4 (100.0) | 5/5 (100.0) | 4/4 (100.0) | 5/5 (100.0) |
| ESBL+ *Enterobacter cloacae* | 0/0 | 0/1 (0.0) | 0/0 | 1/1 (100.0) | 0/0 | 1/1 (100.0) | 0/0 | 1/1 (100.0) |
| *Escherichia coli* | 10/10 (100.0) | 15/15 (100.0) | 10/10 (100.0) | 15/15 (100.0) | 9/9 (100.0) | 14/15 (93.3) | 4/9 (44.4) | 14/15 (93.3) |
| ESBL+ *Escherichia coli* | 4/4 (100.0) | 3/3 (100.0) | 4/4 (100.0) | 3/3 (100.0) | 4/4 (100.0) | 2/3 (66.7) | 0/4 (0.0) | 2/3 (66.7) |
| *Escherichia hermannii* | 0/0 | 1/1 (100.0) | 0/0 | 1/1 (100.0) | 0/0 | 1/1 (100.0) | 0/0 | 0/1 (0.0) |
| ESBL+ *Escherichia hermannii* | 0/0 | 1/1 (100.0) | 0/0 | 1/1 (100.0) | 0/0 | 1/1 (100.0) | 0/0 | 0/1 (0.0) |
| *Klebsiella aerogenes* | 2/3 (66.7) | 2/2 (100.0) | 2/3 (66.7) | 2/2 (100.0) | 0/0 | 0/0 | 0/0 | 0/0 |
| *Klebsiella oxytoca* | 1/1 (100.0) | 4/4 (100.0) | 1/1 (100.0) | 4/4 (100.0) | 1/1 (100.0) | 4/4 (100.0) | 1/1 (100.0) | 4/4 (100.0) |
| ESBL+ *Klebsiella oxytoca* | 0/0 | 2/2 (100.0) | 0/0 | 2/2 (100.0) | 0/0 | 2/2 (100.0) | 0/0 | 2/2 (100.0) |
| *Klebsiella pneumoniae* | 13/32 (40.6) | 21/35 (60.0) | 21/32 (65.6) | 30/35 (85.7) | 25/31 (80.6) | 26/34 (76.5) | 8/31 (25.8) | 12/34 (35.3) |
| ESBL+ *Klebsiella pneumoniae* | 4/23 (17.4) | 7/20 (35.0) | 12/23 (52.2) | 15/19 (78.9) | 17/23 (73.9) | 13/20 (65.0) | 0/23 (0.0) | 0/20 (0.0) |
| *Morganella morganii* | 1/1 (100.0) | 1/1 (100.0) | 1/1 (100.0) | 1/1 (100.0) | 1/1 (100.0) | 1/1 (100.0) | 1/1 (100.0) | 1/1 (100.0) |
| *Pantoea agglomerans* | 1/1 (100.0) | 0/0 | 1/1 (100.0) | 0/0 | 0/0 | 0/0 | 0/0 | 0/0 |
| *Proteus mirabilis* | 1/1 (100.0) | 6/7 (85.7) | 1/1 (100.0) | 7/7 (100.0) | 1/1 (100.0) | 3/6 (50.0) | 0/1 (0.0) | 2/6 (33.3) |
| ESBL+ *Proteus mirabilis* | 1/1 (100.0) | 3/3 (100.0) | 1/1 (100.0) | 3/3 (100.0) | 1/1 (100.0) | 1/3 (33.3) | 0/1 (0.0) | 0/3 (0.0) |
| *Proteus vulgaris* | 0/0 | 1/1 (100.0) | 0/0 | 1/1 (100.0) | 0/0 | 1/1 (100.0) | 0/0 | 1/1 (100.0) |
| *Serratia marcescens* | 3/3 (100.0) | 4/4 (100.0) | 3/3 (100.0) | 4/5 (80.0) | 3/3 (100.0) | 3/3 (100.0) | 2/3 (66.7) | 2/3 (66.7) |
| ESBL+ *Serratia marcescens* | 1/1 (100.0) | 1/1 (100.0) | 1/1 (100.0) | 1/1 (100.0) | 1/1 (100.0) | 1/1 (100.0) | 0/1 (0.0) | 0/1 (0.0) |

|  | **Ceftazidime** | | **Levofloxacin** | | **Piperacillin/tazobactam** | | **Polymyxin B** | |
| --- | --- | --- | --- | --- | --- | --- | --- | --- |
|  | **C/T**  **arm** | **Meropenem arm** | **C/T**  **arm** | **Meropenem arm** | **C/T**  **arm** | **Meropenem**  **arm** | **C/T**  **arm** | **Meropenem**  **arm** |
|  | **n/N1 (%)** | **n/N1 (%)** | **n/N1 (%)** | **n/N1 (%)** | **n/N1 (%)** | **n/N1 (%)** | **n/N1 (%)** | **n/N1 (%)** |
| **Overall** | **29/69 (42.0)** | **56/89 (62.9)** | **27/69 (39.1)** | **54/89 (60.7)** | **36/69 (52.2)** | **65/89 (73.0)** | **58/69 (84.1)** | **75/89 (84.3)** |
| ***Pseudomonas aeruginosa*** | **8/17 (47.1)** | **14/17 (82.4)** | **6/17 (35.3)** | **12/17 (70.6)** | **8/17 (47.1)** | **14/17 (82.4)** | **14/17 (82.4)** | **15/17 (88.2)** |
| **Enterobacterales** | **21/52 (40.4)** | **42/72 (58.3)** | **21/52 (40.4)** | **42/72 (58.3)** | **28/52 (53.8)** | **51/72 (70.8)** | **44/52 (84.6)** | **60/72 (83.3)** |
| ESBL+ Enterobacterales | 1/29 (3.4) | 7/31 (22.6) | 0/29 (0.0) | 6/31 (19.4) | 7/29 (24.1) | 12/31 (38.7) | 25/29 (86.2) | 26/30 (86.7) |
| *Citrobacter freundii* | 0/0 | 0/1 (0.0) | 0/0 | 1/1 (100.0) | 0/0 | 1/1 (100.0) | 0/0 ( | 1/1 (100.0) |
| *Citrobacter koseri* | 2/2 (100.0) | 1/1 (100.0) | 2/2 (100.0) | 1/1 (100.0) | 2/2 (100.0) | 1/1 (100.0) | 2/2 (100.0) | 1/1 (100.0) |
| *Enterobacter cloacae* | 3/4 (75.0) | 4/5 (80.0) | 4/4 (100.0) | 4/5 (80.0) | 3/4 (75.0) | 4/5 (80.0) | 3/4 (75.0) | 5/5 (100.0) |
| ESBL+ *Enterobacter cloacae* | 0/0 | 0/1 (0.0) | 0/0 | 0/1 (0.0) | 0/0 | 0/1 (0.0) | 0/0 | 1/1 (100.0) |
| *Escherichia coli* | 4/9 (44.4) | 14/15 (93.3) | 3/9 (33.3) | 12/15 (80.0) | 8/9 (88.9) | 13/15 (86.7) | 9/9 (100.0) | 15/15 (100.0) |
| ESBL+ *Escherichia coli* | 0/4 (0.0) | 2/3 (66.7) | 0/4 (0.0) | 1/3 (33.3) | 4/4 (100.0) | 1/3 (33.3) | 4/4 (100.0) | 3/3 (100.0) |
| *Escherichia hermannii* | 0/0 | 0/1 (0.0) | 0/0 | 1/1 (100.0) | 0/0 | 1/1 (100.0) | 0/0 | 1/1 (100.0) |
| ESBL+ *Escherichia hermannii* | 0/0 | 0/1 (0.0) | 0/0 | 1/1 (100.0) | 0/0 | 1/1 (100.0) | 0/0 | 1/1 (100.0) |
| *Klebsiella aerogenes* | 0/0 | 0/0 | 0/0 | 0/0 | 0/0 | 0/0 | 0/0 | 0/0 |
| *Klebsiella oxytoca* | 1/1 (100.0) | 4/4 (100.0) | 1/1 (100.0) | 4/4 (100.0) | 1/1 (100.0) | 3/4 (75.0) | 1/1 (100.0) | 4/4 (100.0) |
| ESBL+ *Klebsiella oxytoca* | 0/0 | 2/2 (100.0) | 0/0 | 2/2 (100.0) | 0/0 | 1/2 (50.0) | 0/0 | 2/2 (100.0) |
| *Klebsiella pneumoniae* | 8/31 (25.8) | 10/34 (29.4) | 8/31 (25.8) | 13/34 (38.2) | 10/31 (32.3) | 18/34 (52.9) | 29/31 (93.5) | 33/34 (97.1) |
| ESBL+ *Klebsiella pneumoniae* | 0/23 (0.0) | 0/20 (0.0) | 0/23 (0.0) | 2/20 (10.0) | 2/23 (8.7) | 6/20 (30.0) | 21/23 (91.3) | 19/19 (100.0) |
| *Morganella morganii* | 0/1 (0.0) | 1/1 (100.0) | 1/1 (100.0) | 1/1 (100.0) | 1/1 (100.0) | 1/1 (100.0) | 0/1 (0.0) | 0/1 (0.0) |
| *Pantoea agglomerans* | 0/0 | 0/0 | 0/0 | 0/0 | 0/0 | 0/0 | 0/0 | 0/0 |
| *Proteus mirabilis* | 1/1 (100.0) | 5/6 (83.3) | 0/1 (0.0) | 2/6 (33.3) | 1/1 (100.0) | 6/6 (100.0) | 0/1 (0.0) | 0/6 (0.0) |
| ESBL+ *Proteus mirabilis* | 1/1 (100.0) | 3/3 (100.0) | 0/1 (0.0) | 0/3 (0.0) | 1/1 (100.0) | 3/3 (100.0) | 0/1 (0.0) | 0/3 (0.0) |
| *Proteus vulgaris* | 0/0 ( | 1/1 (100.0) | 0/0 | 1/1 (100.0) | 0/0 | 1/1 (100.0) | 0/0 | 0/1 (0.0) |
| *Serratia marcescens* | 2/3 (66.7) | 2/3 (66.7) | 2/3 (66.7) | 2/3 (66.7) | 2/3 (66.7) | 2/3 (66.7) | 0/3 (0.0) | 0/3 (0.0) |
| ESBL+ *Serratia marcescens* | 0/1 (0.0) | 0/1 (0.0) | 0/1 (0.0) | 0/1 (0.0) | 0/1 (0.0) | 0/1 (0.0) | 0/1 (0.0) | 0/1 (0.0) |
| C/T, ceftolozane/tazobactam. ESBL+, positive for extended spectrum β-lactamase. LRT, lower respiratory tract. n, number of susceptible isolates for the specific pathogen. N1, number of specific pathogen isolates with baseline susceptibility data available. | | | | | | | | |

# Table S4: Baseline microbiologic assessment in ASPECT-NP participants with ventilated hospital-acquired vs those with ventilator-associated bacterial pneumonia (ITT population, both treatment arms combined).

| **Baseline LRT pathogen(s), n (%) *** | **vHABP**  **(N=159)** | **VABP**  **(N=467)** |
| --- | --- | --- |
| **Gram-negative pathogens** | 154 (96.9) | 458 (98.1) |
| *Pseudomonas aeruginosa* | 34 (21.4) | 121 (25.9) |
| AmpC-overexpressing *Pseudomonas aeruginosa* | 4 (2.5) | 16 (3.4) |
| Enterobacterales | 108 (67.9) | 324 (69.4) |
| ESBL+ Enterobacterales | 52 (32.7) | 146 (31.3) |
| *Citrobacter braakii* | 0 | 1 (0.2) |
| *Citrobacter freundii* | 1 (0.6) | 8 (1.7) |
| ESBL+ *Citrobacter freundii* | 0 | 1 (0.2) |
| *Citrobacter koseri* | 5 (3.1) | 8 (1.7) |
| *Enterobacter cloacae* | 9 (5.7) | 26 (5.6) |
| ESBL+ *Enterobacter cloacae* | 1 (0.6) | 1 (0.2) |
| *Enterobacter* sp | 0 | 2 (0.4) |
| *Escherichia coli* | 25 (15.7) | 76 (16.3) |
| ESBL+ *Escherichia coli* | 7 (4.4) | 29 (6.2) |
| *Escherichia hermannii* | 1 (0.6) | 0 |
| ESBL+ *Escherichia hermannii* | 1 (0.6) | 0 |
| *Hafnia alvei* | 0 | 1 (0.2) |
| *Klebsiella (Enterobacter) aerogenes* | 6 (3.8) | 11 (2.4) |
| *Klebsiella oxytoca* | 5 (3.1) | 21 (4.5) |
| ESBL+ *Klebsiella oxytoca* | 2 (1.3) | 1 (0.2) |
| *Klebsiella pneumoniae* | 68 (42.8) | 156 (33.4) |
| ESBL+ *Klebsiella pneumoniae* | 43 (27.0) | 105 (22.5) |
| *Klebsiella* sp | 0 | 1 (0.2) |
| *Morganella morganii* | 2 (1.3) | 8 (1.7) |
| *Pantoea agglomerans* | 1 (0.6) | 1 (0.2) |
| *Proteus mirabilis* | 8 (5.0) | 39 (8.4) |
| ESBL+ *Proteus mirabilis* | 4 (2.5) | 18 (3.9) |
| *Proteus penneri* | 0 | 1 (0.2) |
| *Proteus vulgaris* | 1 (0.6) | 3 (0.6) |
| *Providencia stuartii* | 0 | 2 (0.4) |
| ESBL+ *Providencia stuartii* | 0 | 1 (0.2) |
| *Raoultella ornithinolytica* | 0 | 1 (0.2) |
| *Serratia fonticola* | 0 | 1 (0.2) |
| *Serratia liquefaciens* | 0 | 1 (0.2) |
| *Serratia marcescens* | 8 (5.0) | 24 (5.1) |
| ESBL+ *Serratia marcescens* | 2 (1.3) | 6 (1.3) |
| *Achromobacter* sp | 0 | 1 (0.2) |
| *Achromobacter xylosoxidans* | 3 (1.9) | 2 (0.4) |
| *Acinetobacter baumannii* | 36 (22.6) | 120 (25.7) |
| *Acinetobacter calcoaceticus* | 1 (0.6) | 1 (0.2) |
| *Acinetobacter junii* | 0 | 1 (0.2) |
| *Acinetobacter lwoffii* | 2 (1.3) | 0 |
| *Acinetobacter* sp | 1 (0.6) | 3 (0.6) |
| *Aeromonas hydrophila* | 1 (0.6) | 1 (0.2) |
| *Aggregatibacter segnis* | 0 | 1 (0.2) |
| *Alcaligenes faecalis* | 0 | 1 (0.2) |
| *Burkholderia cepacia* | 2 (1.3) | 1 (0.2) |
| *Burkholderia gladioli* | 0 | 1 (0.2) |
| *Burkholderia multivorans* | 0 | 1 (0.2) |
| *Burkholderia vesicularis* | 1 (0.6) | 0 |
| *Delftia acidovorans* | 1 (0.6) | 0 |
| *Elizabethkingia meningosepticum* | 0 | 1 (0.2) |
| *Haemophilus haemolyticus* | 0 | 1 (0.2) |
| *Haemophilus influenzae* | 9 (5.7) | 32 (6.9) |
| *Haemophilus parainfluenzae* | 0 | 1 (0.2) |
| *Haemophilus* sp | 1 (0.6) | 0 |
| *Moraxella catarrhalis* | 0 | 1 (0.2) |
| *Moraxella* sp | 0 | 1 (0.2) |
| *Neisseria meningitidis* | 1 (0.6) | 0 |
| *Neisseria* sp | 0 | 1 (0.2) |
| *Pseudomonas putida* | 0 | 4 (0.9) |
| *Stenotrophomonas maltophilia* | 4 (2.5) | 7 (1.5) |
| **Gram-positive pathogens** | 17 (10.7) | 25 (5.4) |
| *Streptococcus agalactiae* | 1 (0.6) | 1 ( .2) |
| *Streptococcus constellatus* | 0 | 2 ( .4) |
| *Streptococcus milleri* | 0 | 1 ( .2) |
| *Streptococcus mitis* | 1 (0.6) | 3 ( .6) |
| *Streptococcus parasanguinis* | 0 | 1 (0.2) |
| *Streptococcus pneumoniae* | 7 (4.4) | 8 (1.7) |
| *Streptococcus* sp, alpha hemolytic | 3 (1.9) | 4 (0.9) |
| *Streptococcus* sp, beta hemolytic | 2 (1.3) | 5 (1.1) |
| *Streptococcu*s sp, non-hemolytic | 2 (1.3) | 0 |
| *Streptococcus viridans* | 2 (1.3) | 2 (0.4) |

N, number of ITT population patients in the respective subpopulation. n, number of patients with the respective pathogen.

^*^Participants with more than 1 pathogen isolated at baseline are counted only once within each pathogen category.

# Table S5: Baseline bacterial pathogen isolates from blood cultures in the ventilated HABP subgroup, by treatment arm (ITT population).

| **Pathogen** | **C/T** | **Meropenem** | **Total** |
| --- | --- | --- | --- |
|  | **(N=14)** | **(N=11)** | **(N=25)** |
| Number of Participants with ≥1 Blood Isolate | 14 (100%) | 11 (100%) | 25 (100%) |
| Gram-Negative Pathogens | 6 (42.9%) | 7 (63.6%) | 13 (52.0%) |
| *Acinetobacter baumannii* | 4 (28.%6) | 2 (18.2%) | 6 (24.0%) |
| *Enterobacter cloacae* | 0 | 2 (18.2%) | 2 (8.0%) |
| *Klebsiella pneumoniae* | 3 (21.4%) | 2 (18.2%) | 5 (20.0%) |
| *Morganella morganii* | 0 | 1 (9.1%) | 1 (4.0%) |
| *Pseudomonas fluorescens* | 0 | 1 (9.1%) | 1 (4.0%) |
| Gram-Positive Pathogens | 9 (64.3%) | 4 (36.4%) | 13 (52.0%) |
| Coagulase negative *Staphylococcus* | 0 | 1 (9.1%) | 1 (4.0%) |
| *Enterococcus faecalis* | 2 (14.3%) | 0 | 2 ( 8.0%) |
| *Staphylococcus aureus* | 1 (7.1%) | 0 | 1 (4.0%) |
| *Staphylococcus chromogenes* | 0 | 1 (9.1%) | 1 (4.0%) |
| *Staphylococcus cohnii* | 1 (7.1%) | 0 | 1 (4.0%) |
| *Staphylococcus epidermidis* | 5 (35.7%) | 1 (9.1%) | 6 (24.0%) |
| *Staphylococcus hominis* | 3 (21.4%) | 2 (18.2%) | 5 (20.0%) |
| *Staphylococcus hyicus* | 1 (7.1%) | 0 | 1 (4.0%) |
| *Staphylococcus lentus* | 1 (7.1%) | 1 (9.1%) | 2 (8.0%) |
| *Streptococcus anginosus* | 1 (7.1%) | 0 | 1 (4.0%) |
| *Streptococcus gordonii* | 1 (7.1%) | 0 | 1 (4.0%) |
| C/T, ceftolozane/tazobactam. n, Number of participants in specific category. N, Number of participants in population.  Percentages are calculated as 100 x (n/N). | | | |

# Table S6: 28-day all-cause mortality in participants with vHABP, by clinical characteristics evaluated in the multivariable analysis (ITT population).

| **Clinical characteristic** | **Subgroup** | **C/T** | **Meropenem** | **% Difference** |  |
| --- | --- | --- | --- | --- | --- |
|  |  | **n/N (%)** | **n/N (%)** | **(95% CI)** |  |
| **Overall** | - | 24 / 99 (24.2) | 40 / 108 (37.0) | 12.8 (0.2, 24.8) |  |
| **Baseline Age** | <65 years | 10 / 52 (19.2) | 16 / 47 (34.0) | 14.8 (-2.6, 31.4) |  |
|  | ≥65 years | 14 / 47 (29.8) | 24 / 61 (39.3) | 9.6 (-8.6, 26.3) |  |
| **Baseline CrCL (mL/min) Category** | ≥80 mL/min (normal) | 10 / 44 (22.7) | 16 / 48 (33.3) | 10.6 (-7.8, 27.9) |  |
|  | >50 - <80 mL/min (mild impairment) | 7 / 26 (26.9) | 12 / 34 (35.3) | 8.4 (-15.2, 29.7) |  |
|  | ≥30 - ≤50 mL/min (moderate impairment) | 4 / 17 (23.5) | 4 / 10 (40.0) | 16.5 (-16.7, 48.4) |  |
|  | ≥15 - <30 mL/min (severe impairment) | 3 / 12 (25.0) | 7 / 13 (53.8) | 28.8 (-8.7, 56.9) |  |
|  | ≥150 mL/min (hyperclearance) | 3 / 9 (33.3) | 2 / 5 (40.0) | NC |  |
| **Failure of Prior Antibiotic Therapy for Current vHABP Episode** | Yes | 7 / 20 (35.0) | 7 / 17 (41.2) | 6.2 (-23.1, 34.6) |  |
|  | No | 17 / 79 (21.5) | 33 / 90 (36.7) | 15.1 (1.4, 28.0) |  |
| **Baseline APACHE-II Score** | <20 | 13 / 68 (19.1) | 22 / 67 (32.8) | 13.7 (-1.1, 27.8) | |
|  | ≥20 | 11 / 31 (35.5) | 17 / 40 (42.5) | 7.0 (-15.4, 28.0) | |
| **Baseline Bacteremia (Any Pathogen)** | Yes | 5 / 14 (35.7) | 8 / 11 (72.7) | 37.0 (-1.8, 63.1) | |
|  | No | 19 / 85 (22.4) | 32 / 97 (33.0) | 10.6 (-2.5, 23.1) | |
| **Baseline Gram-negative Adjunctive Therapy** | Yes | 10 / 35 (28.6) | 14 / 34 (41.2) | 12.6 (-9.6, 33.2) | |
|  | No | 14 / 64 (21.9) | 25 / 70 (35.7) | 13.8 (-1.6, 28.2) | |
| **Baseline CPIS** | ≤6 | 4 / 9 (44.4) | 2 / 7 (28.6) | -15.9 (-51.2, 27.9) | |
|  | =7 | 3 / 12 (25.0) | 4 / 7 (57.1) | 32.1 (-10.6, 63.6) | |
|  | =8 | 1 / 13 (7.7) | 3 / 13 (23.1) | 15.4 (-14.3, 43.3) | |
|  | >8 | 16 / 65 (24.6) | 31 / 80 (38.8) | 14.1 (-1.2, 28.2) | |
| **Baseline PaO_2_/FiO_2_ (mmHg)** | ≤240 | 18 / 75 (24.0) | 30 / 79 (38.0) | 14.0 (-0.7, 27.7) | |
|  | >240 | 6 / 23 (26.1) | 9 / 27 (33.3) | 7.2 (-17.9, 30.5) | |
| **Baseline SOFA Score** | ≤7 | 13 / 57 (22.8) | 19 / 65 (29.2) | 6.4 (-9.3, 21.4) | |
|  | >7 | 11 / 42 (26.2) | 20 / 41 (48.8) | 22.6 (1.8, 40.9) | |
| **Duration of Hospitalization Prior to Randomization** | <5 days | 8 / 27 (29.6) | 11 / 32 (34.4) | 4.7 (-18.7, 26.9) | |
|  | ≥5 days | 16 / 71 (22.5) | 28 / 72 (38.9) | 16.4 (1.2, 30.5) | |
| **Duration of Mechanical Ventilation Prior to Randomization** | <5 days | 22 / 88 (25.0) | 32 / 93 (34.4) | 9.4 (-3.9, 22.21) | |
|  | ≥5 days | 2 / 11 (18.2) | 7 / 12 (58.3) | 40.2 (0.6, 66.0) | |
| **Vasopressor Use** | Yes | 19 / 55 (34.5) | 30 / 55 (54.5) | 20.0 (1.5, 36.7) | |
|  | No | 5 / 44 (11.4) | 10 / 53 (18.9) | 7.5 (-7.6, 21.5) | |
| **Baseline *Pseudomonas aeruginosa*** | Yes | 2 / 17 (11.8) | 5 / 17 (29.4) | 17.6 (-10.1, 42.8) | |
|  | No | 22 / 82 (26.8) | 35 / 91 (38.5) | 11.6 (-2.4, 24.9) | |
| **Baseline ESBL-positive Enterobacterales** | Yes | 9 / 27 (33.3) | 11 / 25 (44.0) | 10.7 (-14.9, 34.6) | |
|  | No | 15 / 72 (20.8) | 29 / 83 (34.9) | 14.1 (-0.2, 27.4) | |

CI, confidence interval. CPIS, clinical pulmonary infection score. CrCL, creatinine clearance. C/T, ceftolozane/tazobactam. ESBL, extended-spectrum β-lactamase. N, number of ITT patients in vHAP subpopulation per treatment arm. NC, not calculated (due to small sample sizes). ITT, intent to treat population (all randomized patients). TOC, test of cure. vHABP, ventilated hospital-acquired bacterial pneumonia.

# Table S7: Odds ratio estimates (and confidence intervals) for risk of death due to any cause by day 28 associated with the significant factors in the sensitivity logistic regression model.

| **Patient characteristic** | **Odds ratio for 28-day ACM^a^**  **(95% CI)** |
| --- | --- |
| Baseline bacteremia with any pathogen (vs no bacteremia)^a^ | 5.3  (1.7, 16.6) |
| Concomitant vasopressor use (vs no vasopressor use)^b^ | 6.2  (2.6, 14.7) |
| Meropenem treatment (versus ceftolozane/tazobactam treatment)^c^ | 2.7  (1.3, 5.8) |

ACM, all-cause mortality. CI, confidence interval.

^a^Adjusting for all 16 factors.

# Figure S1. Methodology and general results of the multivariable analysis.

Variable selection for the final backward elimination logistic main effects regression model was done through forward selection stepwise regression (the order in which explanatory variables were entered into forward selection was determined by their relative ranking of importance from a random forest analysis).
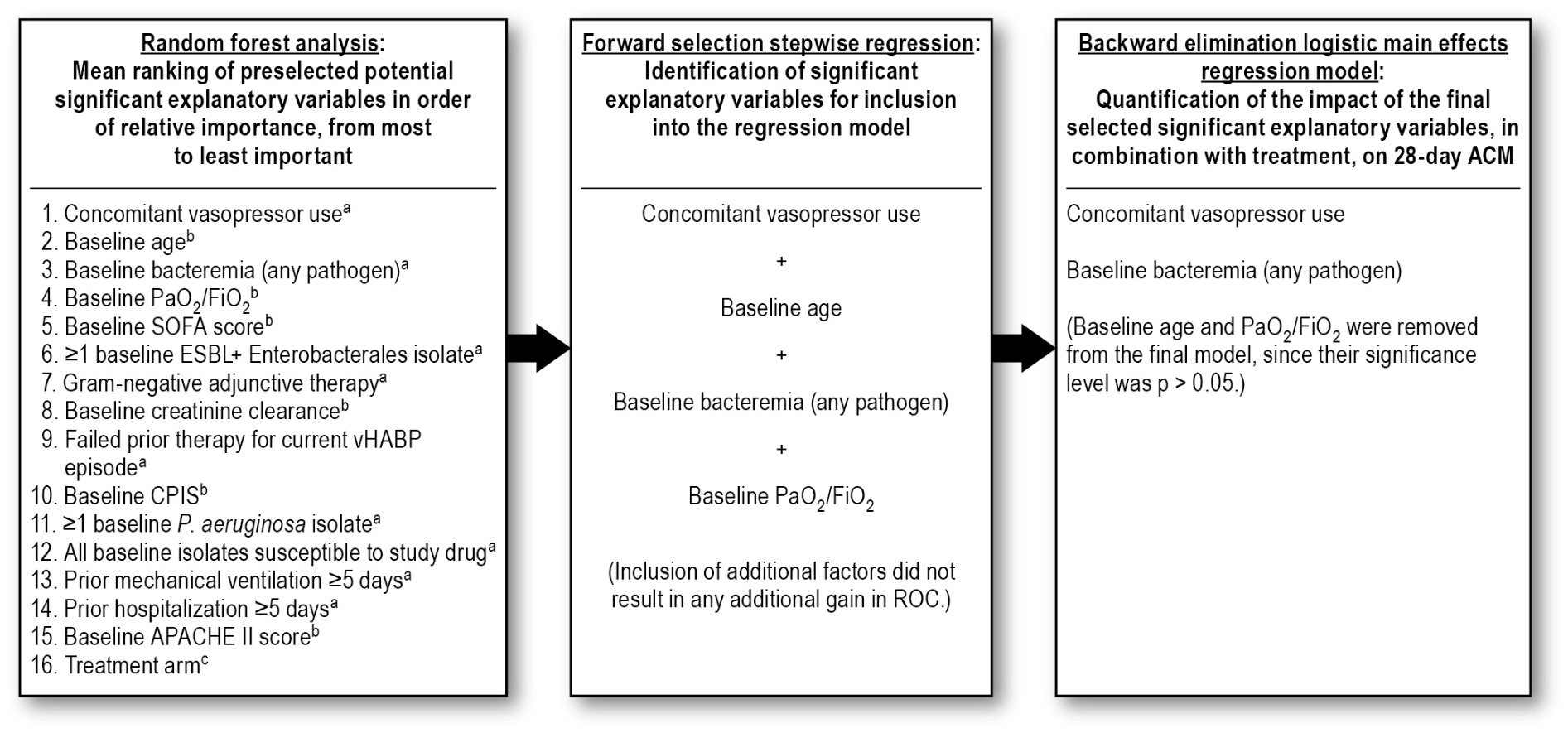
^a^Dichotomous variable. ^b^Continuous variable. ^c^Dichotomous variable; treatment group was to be included into the final regression model irrespective of results from previous steps, since the goal was to determine the impact of treatment assignment on mortality relative to the impact of the most influential factors.

ACM, all-cause mortality. APACHE, acute physiology and chronic health evaluation. CPIS, clinical pulmonary infection score. ESBL, extended-spectrum β-lactamase. PaO_2_/FiO_2_, ratio of arterial oxygen partial pressure to fractional inspired oxygen. ROC, area under the receiver operated curve. SOFA, sequential organ failure assessment. vHABP, ventilated hospital-acquired bacterial pneumonia.

# Figure S2. Frequency distribution of meropenem MIC values for (A) Enterobacterales (N=80 isolates) and (B) *Pseudomonas aeruginosa* (N=19) isolates obtained from participants with vHABP of the meropenem arm. Breakpoints indicated reflect current (2021) CLSI susceptibility breakpoints for meropenem.

(**A**) Enterobacterales (CLSI susceptibility breakpoint for meropenem: 1 μg/mL)


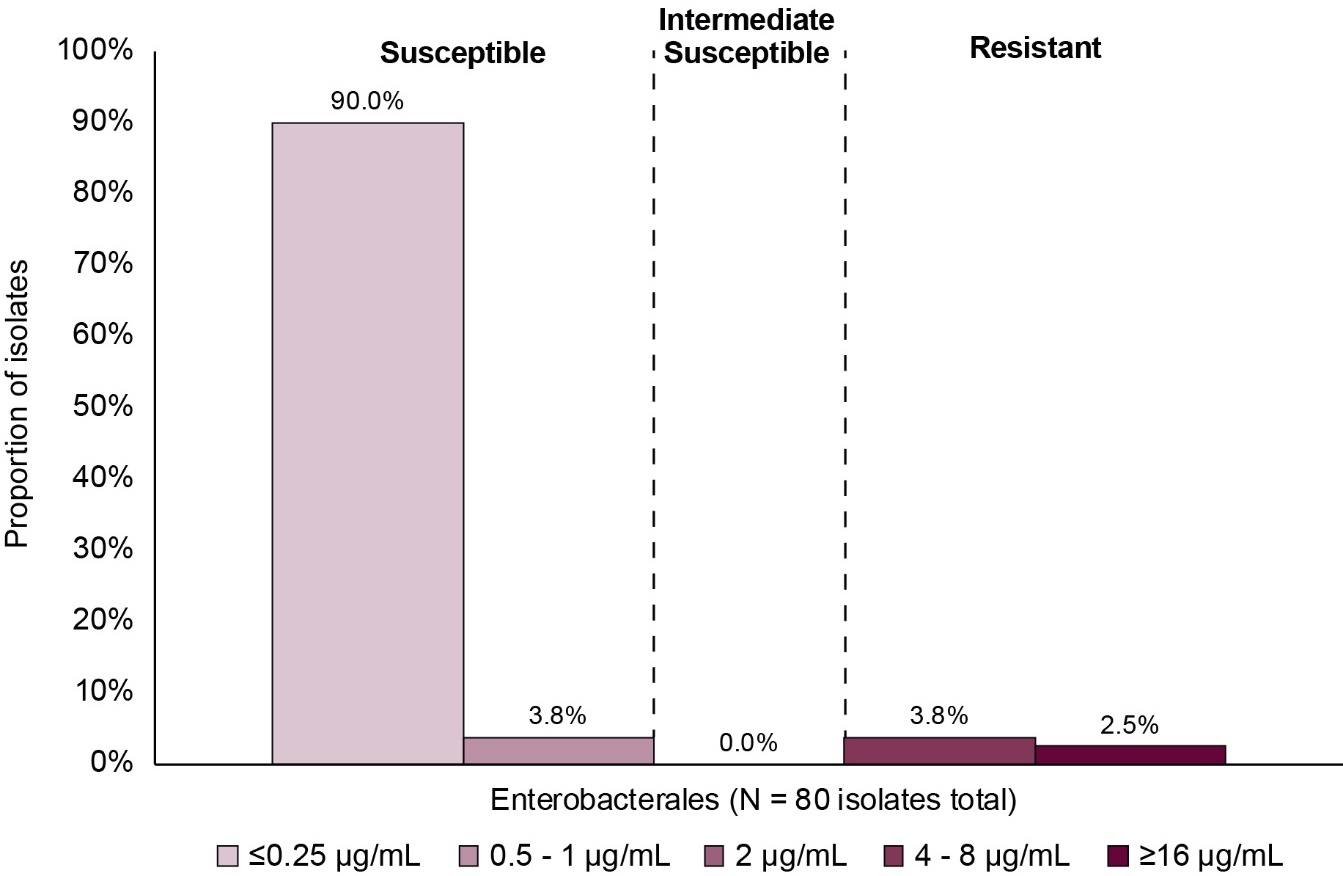


(**B**) *Pseudomonas aeruginosa* (CLSI susceptibility breakpoint for meropenem: 2 μg/mL)


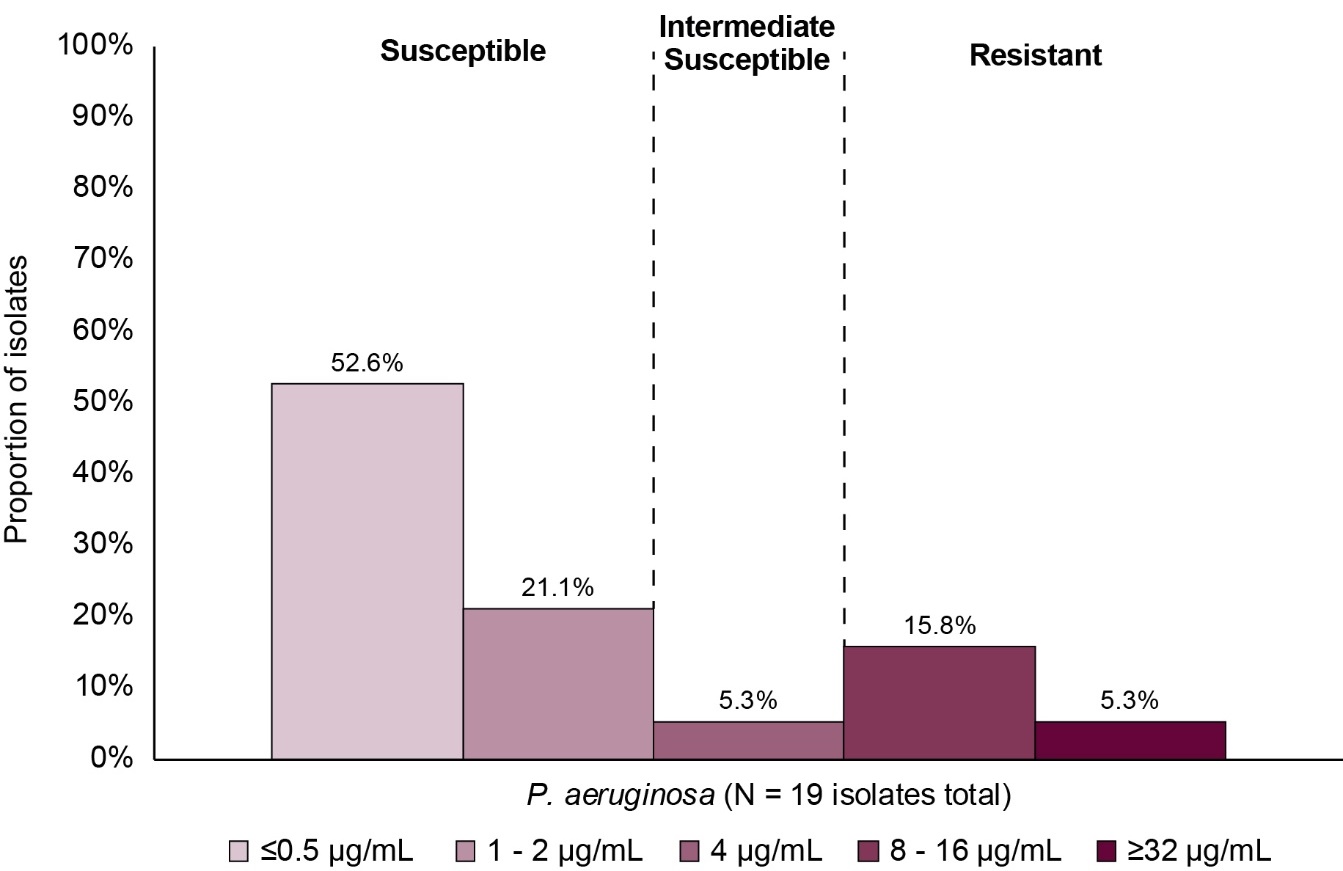

Supplement: Supplementary file 1 — Additional file 1: Table S1. Baseline demographics and clinical characteristics in ASPECT-NP participants with ventilated hospital-acquired vs those with ventilator-associated bacterial pneumonia (ITT population, both treatment arms combined). Table S2. Baseline microbiologic assessment in ASPECT-NP participants with ventilated hospital-acquired bacterial pneumonia (ITT population). Table S3. Susceptibility of baseline Pseudomonas aeruginosa and Enterobacterales to key antibacterial agents, by treatment arm, in ASPECT-NP participants with ventilated hospital-acquired bacterial pneumonia (ITT population). Table S4. Baseline microbiologic assessment in ASPECT-NP participants with ventilated hospital-acquired vs those with ventilator-associated bacterial pneumonia (ITT population, both treatment arms combined). Table S5. Baseline bacterial pathogen isolates from blood cultures in the ventilated HABP subgroup, by treatment arm (ITT population). Table S6. 28-day all-cause mortality in participants with vHABP, by clinical characteristics evaluated in the multivariable analysis (ITT population). Table S7. Odds ratio estimates (and confidence intervals) for risk of death due to any cause by day 28 associated with the significant factors in the sensitivity logistic regression model. Figure S1. Methodology and general results of the multivariable analysis. Figure S2. Frequency distribution of meropenem MIC values for (A) Enterobacterales (N = 80 isolates) and (B) Pseudomonas aeruginosa (N = 19) isolates obtained from participants with vHABP of the meropenem arm. [file 13054_2021_3694_MOESM1_ESM.docx]
